# Drosophila *Nipped-B* Mutants Model Cornelia de Lange Syndrome in Growth and Behavior

**DOI:** 10.1371/journal.pgen.1005655

**Published:** 2015-11-06

**Authors:** Yaning Wu, Maria Gause, Dongbin Xu, Ziva Misulovin, Cheri A. Schaaf, Ramya C. Mosarla, Elizabeth Mannino, Megan Shannon, Emily Jones, Mi Shi, Wen-Feng Chen, Olivia L. Katz, Amita Sehgal, Thomas A. Jongens, Ian D. Krantz, Dale Dorsett

**Affiliations:** 1 Division of Human Genetics, The Children's Hospital of Philadelphia, Philadelphia, Pennsylvania, United States of America; 2 Edward A Doisy Department of Biochemistry and Molecular Biology, Saint Louis University School of Medicine, Saint Louis, Missouri, United States of America; 3 Howard Hughes Medical Institute and Department of Neuroscience, Perelman School of Medicine at the University of Pennsylvania, Philadelphia, Pennsylvania, United States of America; 4 Department of Genetics, Perelman School of Medicine at the University of Pennsylvania, Philadelphia, Pennsylvania, United States of America; University of California, Irvine, UNITED STATES

## Abstract

Individuals with Cornelia de Lange Syndrome (CdLS) display diverse developmental deficits, including slow growth, multiple limb and organ abnormalities, and intellectual disabilities. Severely-affected individuals most often have dominant loss-of-function mutations in the *Nipped-B-Like* (*NIPBL*) gene, and milder cases often have missense or in-frame deletion mutations in genes encoding subunits of the cohesin complex. Cohesin mediates sister chromatid cohesion to facilitate accurate chromosome segregation, and NIPBL is required for cohesin to bind to chromosomes. Individuals with CdLS, however, do not display overt cohesion or segregation defects. Rather, studies in human cells and model organisms indicate that modest decreases in NIPBL and cohesin activity alter the transcription of many genes that regulate growth and development. Sister chromatid cohesion factors, including the Nipped-B ortholog of NIPBL, are also critical for gene expression and development in *Drosophila melanogaster*. Here we describe how a modest reduction in Nipped-B activity alters growth and neurological function in Drosophila. These studies reveal that *Nipped-B* heterozygous mutant Drosophila show reduced growth, learning, and memory, and altered circadian rhythms. Importantly, the growth deficits are not caused by changes in systemic growth controls, but reductions in cell number and size attributable in part to reduced expression of *myc* (*diminutive*) and other growth control genes. The learning, memory and circadian deficits are accompanied by morphological abnormalities in brain structure. These studies confirm that *Drosophila Nipped-B* mutants provide a useful model for understanding CdLS, and provide new insights into the origins of birth defects.

## Author Summary

Cornelia de Lange Syndrome (CdLS) alters many aspects of growth and development. CdLS is caused by mutations in genes encoding proteins that ensure that chromosomes are distributed equally when a cell divides. These include genes that encode components of the cohesin complex, and Nipped-B-Like (NIPBL) that puts cohesin onto chromosomes. Individuals with CdLS have only modest reductions in the activities of these genes and do not show changes in chromosome distribution. Instead, they show differences in the expression many genes that control development. Animal models of CdLS will be useful for studies aimed at understanding how development is altered, and testing methods for treating CdLS. We find that Drosophila with one mutant copy of the *Nipped-B* gene, which is equivalent to the *NIPBL* gene, show characteristics similar to individuals with CdLS. These include reduced growth, learning, memory, and altered circadian rhythms. These studies thus indicate that Drosophila *Nipped-B* mutants are a valuable system for investigating the causes of the CdLS birth defects, and developing potential treatments. They also reveal that the slow growth in Drosophila *Nipped-B* mutants is not caused by disruption of systemic hormonal growth controls, and that the learning and memory deficits may reflect changes in brain structure.

## Introduction

The ring-like cohesin complex consisting of the Smc1, Smc3, Stromalin (SA) and Rad21 proteins is required for sister chromatid cohesion and accurate chromosome segregation. Cohesin is loaded on to chromosomes by the kollerin complex, consisting of the NIPBL adherin (Nipped-B in Drosophila) and Mau-2 proteins, and is removed from chromosomes by the releasin complex containing the Wapl and Pds5 proteins [1–4]. Adherin, cohesin and releasin also function in gene transcription in a dosage-sensitive manner. Reductions in their activities alter the expression of hundreds of genes that control growth and development in metazoan organisms. These changes in gene expression occur in the absence of detectable faults in sister chromatid cohesion or chromosome segregation, and are accompanied by defects in development in Drosophila, zebrafish, mice and humans [5–20].

Adherin and cohesin regulate transcription via multiple mechanisms. They bind primarily to active genes and transcriptional enhancers, with high levels near the transcription start sites of the active genes [15,21–24]. Substantial genetic and molecular evidence supports the idea that cohesin facilitates enhancer-promoter communication. In Drosophila, the genes that bind cohesin have high levels of transcriptional pausing, in which RNA polymerase II (Pol II) stops after transcribing some 30 to 40 nucleotides, but remains transcriptionally engaged [24,25]. Adherin and cohesin can both facilitate and inhibit transition of the paused Pol II into elongation, depending on the specific gene and cell type [24,25]. Although the association of cohesin with transcriptional pausing has not been systematically explored in mammalian cells, many NIPBL- and cohesin-binding genes, such as *c-myc*, display high transcriptional pausing. In addition to direct control of active gene transcription, evidence from Drosophila shows that cohesin indirectly controls epigenetic silencing of homeotic genes by Polycomb complexes via sequestration of the PRC1 Polycomb complex at active genes and limiting the amount available for silencing [4]. It is currently unknown if cohesin plays a similar role in mammalian Polycomb silencing.

In humans, dominant loss-of-function mutations in *NIPBL* and missense or in-frame deletion mutations in the *SMC1A* and *SMC3* cohesin subunit genes cause a striking constellation of structural and intellectual birth defects called Cornelia de Lange syndrome (CdLS, reviewed in [26,27]). Mutations in the *RAD21* cohesin subunit gene, and in the *HDAC8* gene encoding a deacetylase that recycles SMC3 cause a very similar set of developmental deficiencies [20,28]. These closely-related birth defect syndromes are known collectively as cohesinopathies. Individuals with classical CdLS exhibit clinical phenotypes affecting multiple organs and systems, such as characteristic craniofacial dysmorphia, pre- and postnatal growth and developmental delay, microcephaly, intellectual disability, musculoskeletal defects and diverse organ system involvement. However, mildly affected individuals may present with only mild cognitive and growth retardation and lack overt structural abnormalities [27,29].

A clinical diagnosis of CdLS is usually made by recognition of the characteristic facial features, which include arched eyebrows, long philtrum, thin lips, and low set back ears [30]. While these craniofacial features are present in 95% of classic CdLS, they can be quite subtle in milder cases. Importantly, growth reduction is the most common finding in CdLS, and may be the main clinical presentations in mild cases [11,20]. Intellectual disability and learning differences are also universally seen in individuals with CdLS, with the average IQ score being 53, (range of 30 to 100) [27]. *NIPBL* heterozygous mutations are present in nearly 60% of CdLS individuals and mutations in cohesin subunits and *HDAC8* account for an additional 10–15% of cases overall [27,31]. Individuals with *NIPBL* mutations tend to be more severely affected, with slow growth, upper limb defects and organ defects and severe intellectual disabilities, while those with cohesin subunit mutations tend to have less severe growth and structural birth defects. Genotype-phenotype correlations among a large number of *NIPBL* probands indicate that *NIPBL* haploinsufficiency, resulting from nonsense mutations, splice site mutations, or frame-shift deletions/insertions, generally leads to more severe cognitive and structural phenotypes than missense mutations [32].

Understanding the origins of the developmental deficits in the cohesinopathies is complicated by their diversity, and the findings that many genes encoding components of all major signaling pathways, and transcription factors that control growth and development, such as c-Myc and HOX proteins, are bound and transcriptionally-influenced by adherin and cohesin. Much of the current understanding of how adherin and cohesin modulate transcription comes from Drosophila, due to its less complex genome and availability of substantial genetic tools. The evidence that many of the same genes and signaling pathways are regulated by adherin and cohesin in Drosophila and vertebrates raises the possibility that Drosophila could also provide fundamental insights into the developmental origins of many of the birth defects observed in human cohesinopathies. Heterozygous null mutant *Nipped-B* Drosophila do not appear to be as strongly affected as humans, and require weak or cryptic mutations in other genes to display overt structural alterations [5,17,33–35]. Due to the almost universal presence of cognitive, learning and growth deficits in humans with CdLS we decided to more closely examine *Nipped-B* mutant Drosophila for these specific phenotypes. These studies reveal that *Nipped-B* mutant *Drosophila* also display substantial growth and neurological phenotypes. Strikingly, the data indicate that there are no appreciable changes in systemically controlled developmental timing or growth regulation. The *Nipped-B* mutant growth and intellectual phenotypes largely correlate with changes in cell number, size and morphology.

## Results

### Heterozygous *Nipped-B* mutations reduce growth to a similar extent as mutations in the *dm* (*myc*) and *Tor* growth regulators

Individuals with CdLS are usually small for their age, but the reasons for their reduced size are largely unknown, and could potentially reflect altered systemic growth control, delayed developmental timing, reduced cell division, reduced utilization of nutrition, increased cell death, or any combination of these factors. We find that heterozygous *Nipped-B* loss-of-function mutant (*Nipped-B* ^*407*^ / +) adult male and female Drosophila weigh 5 to 8% less than matched wild-type flies, which represent significant decreases, similar in magnitude to those caused by heterozygous null mutations in the *diminutive* (*dm*, *myc*) and *Tor* (*Target of rapamycin*) growth regulators (Table 1). For all experiments, the *Nipped-B* ^*407*^ and P57B wild-type parental control chromosomes were compared after outcrossing to a wild-type Oregon R background. We chose outcrossing to a wild-type strain to minimize potential genetic background effects on growth. Outcrossing ensures that the background is heteroallelic for wild-type alleles across the genome, in contrast to inbred isogenic backgrounds, which tend to harbor recessive genetic modifiers of growth, viability and fertility.

**Table 1:** Effects of heterozygous loss-of-function *Nipped-B*, *dm*, and *Tor* mutations on adult body weight.

	Control adult weight (mg per fly)				Ratio of mutant weight to matched control weight					
Food (%)	P57B		*y w*		*Nipped-B* ^*407*^		*dm* ^*2*^		*Tor* ^*ΔP*^	
	♂	♀	♂	♀	♂	♀	♂	♀	♂	♀
100	0.88	1.37	0.91	1.33	0.95	0.92	na	0.86	0.96	0.92
	± 0.01^a^	± 0.01	± 0.01	± 0.01	p = 0.0008^b^	p<0.0001		p<0.0001	p = 0.02	p = 0.0004
20	0.60	0.89	0.60	0.85	0.99	0.94	na	0.91	0.99	0.95
	± 0.01	± 0.02	± 0.02	± 0.01	p = 0.6	p = 0.04		p = 0.002	p = 0.7	p = 0.1

^a^Standard error.

^b^P values determined using the student t test.

The *myc* gene (Drosophila *diminutive*, *dm*) encodes a transcription factor that promotes protein synthesis, cell growth and division. It is positively regulated by cohesin in Drosophila, zebrafish, mouse and human [14,15,17,37]. Female heterozygous mutant for a strong loss-of-function *dm* allele (*dm* ^*2*^) showed a weight reduction of 14%, greater than that observed in *Nipped-B* mutants (Table 1). We could not measure the effects in males because *dm* is X-linked, and the *dm* ^*2*^ allele is lethal. *dm* ^*2*^ is a point mutation that causes a nonsense mutation and behaves similar to null alleles [38].

The Target of rapamycin (Tor) kinase integrates insulin and growth factor signaling to promote protein synthesis, growth and cell proliferation, and influences the expression of many of the same genes as *dm* [39,40]. Heterozygous *Tor* null mutant (*Tor* ^*ΔP*^) male and female adults showed 4 to 8% reduced weight, similar to *Nipped-B* mutant flies (Table 1). *Tor* ^*ΔP*^ is a 3.5 kb deletion created by a P element transposon excision that removes the translation start site [41]. Because we do not have the parental chromosomes for the *dm* and *Tor* mutations, and both were generated in a *y w* background [38,41] we used a *y w* stock as the wild-type control for both *dm* ^*2*^ and *Tor* ^*ΔP*^. As with the *Nipped-B* ^*407*^ and matched P57B control, the *y w* control and the *dm* and *Tor* mutants were compared after outcrossing into a wild-type Oregon R background to reduce the likelihood of recessive growth modifiers.

We carefully chose the *Nipped-B* ^*407*^ allele as optimal for close examination of growth phenotypes because in contrast to all other available mutant alleles, there is substantial evidence that it is functionally null and lacks other linked mutations. *Nipped-B* is a large gene (~40 kb) located in centromere-proximal heterochromatin on chromosome 2R in a region refractory to meiotic recombination (S1 Fig). The introns and flanking sequences consist primarily of natural transposons, making it challenging to molecularly characterize *Nipped-B* mutations, and to ensure that there are no other linked mutations. When it was isolated, *Nipped-B* ^*407*^ was backcrossed multiple times to the parental P57B wild-type chromosome and extensively tested for lack of allelism to all known essential genes in the surrounding heterochromatin [5]. Genomic sequencing and comparison to RNA-seq data indicates that *Nipped-B* ^*407*^ is a regulatory mutation that produces little or no RNA (S1 and S2 Figs) in contrast to the sequenced chemically-induced alleles that produce missense or truncated proteins [33]. As described in detail below, *Nipped-B* ^*407*^ dominantly reduces wing growth to a similar extent as two other mutant *Nipped-B* alleles, *Nipped-B* ^*292*.*1*^ and *T(2;3)Nipped-B* ^*359*.*1*^. Compared to other mutant alleles, *Nipped-B* ^*407*^ usually has strong dominant genetic interactions with mutations in other genes [5,33]. Crucially, *Nipped-B* ^*407*^ is the only *Nipped-B* allele shown to be the only recessive lethal mutation on its chromosome by rescuing survival beyond the normal 2^nd^ instar lethal phase to adults or pharate adults with cDNA transgenes [36,42]. It is likely that cDNA transgenes do not fully rescue viability because of the need to use a foreign promoter, and the inability to produce the several N terminal splice variants [33] (S2 Fig).

Drosophila grown under limited nutrition (20% of the normal food concentration) showed 30 to 40% reductions in weight, which is substantially greater than the weight loss caused by the heterozygous *Nipped-B*, *dm*, or *Tor* mutations (Table 1). We reasoned that if *Nipped-B*, *dm* or *Tor* mutations decrease the ability of the flies to utilize the available nutrients to support growth, then these mutants might show a greater sensitivity to reduced nutrition than wild-type. Opposite to this expectation, *Nipped-B* and *Tor* mutant males showed the same sensitivity as wild-type to reduced nutrition, and weighed essentially the same as wild-type flies grown under the limiting conditions (Table 1). Similarly, *Nipped-B*, *dm*, and *Tor* mutant females showed a less significant reduction in weight relative to wild-type under limiting nutrition. In other words, restricting nutrition partially suppressed and did not enhance the relative growth deficits in *Nipped-B*, *dm*, and *Tor* mutants. Thus the primary growth deficit in *Nipped-B* mutants does not stem from decreased utilization of available food.

### Systemic growth control and developmental timing are not detectably altered in heterozygous *Nipped-B* mutants

We tested if the reduced growth of *Nipped-B*, *dm* and *Tor* mutant flies correlates with altered developmental timing, or altered systemic growth controls that normally delay hormonally-driven developmental transitions until the organism reaches a critical size [43]. We collected newly-hatched larvae and measured the times from hatching to pupariation and to eclosion of adults when grown on normal and 20% food. With normal systemic growth controls, reducing nutrition delays the developmental transitions.

**Fig 1:**
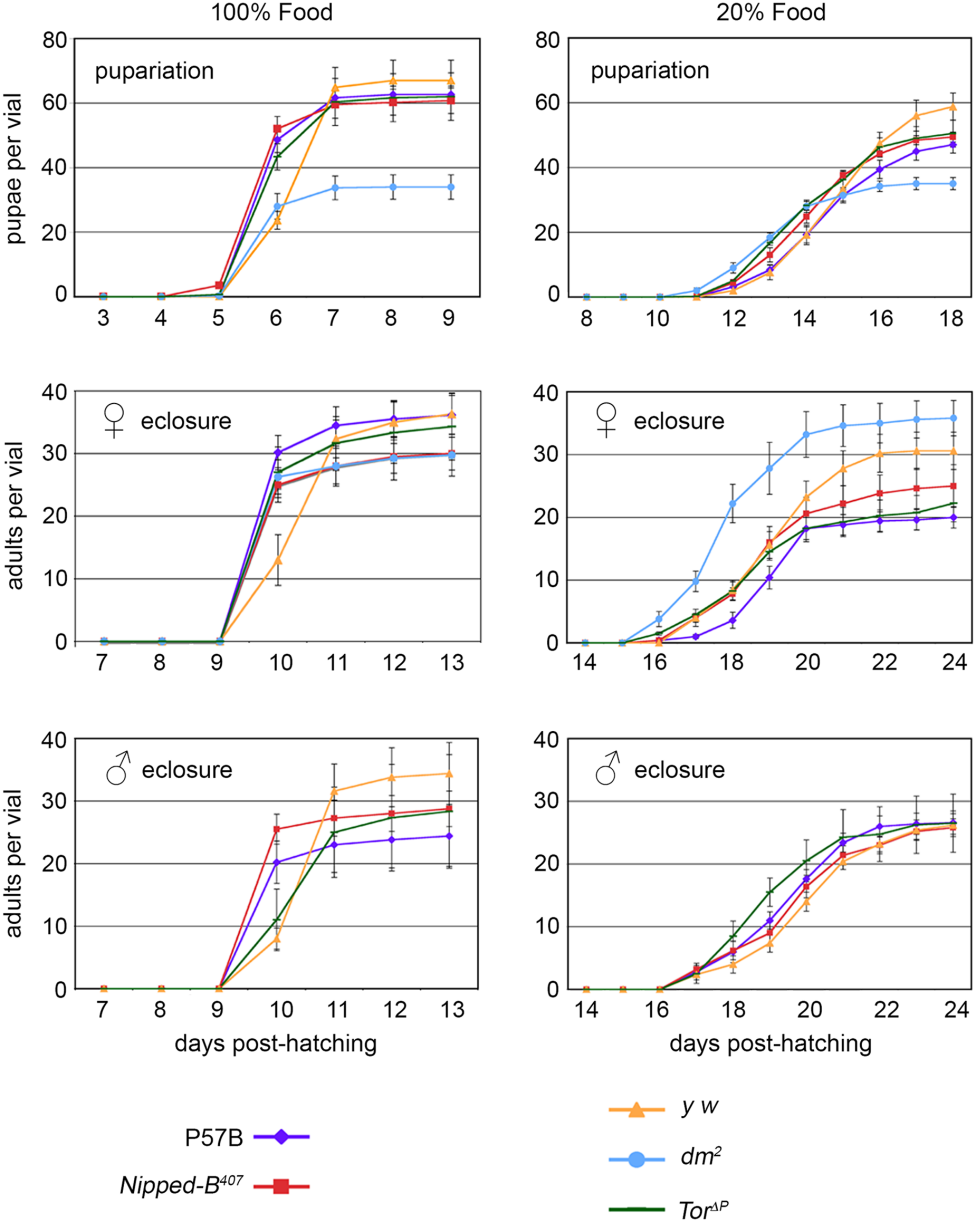
Effects of heterozygous *Nipped-B*, *dm*, and *Tor* mutations on timing of pupariation and eclosion. Newly hatched 1^st^ instar larvae of the indicated genotypes were placed in vials at 100 per vial, containing either normal (100%) or diluted food (20%). The number of pupae and eclosing male and female adults were counted every day for up to 24 days. P57B (P57B/+) is the matched wild-type parental control for *Nipped-B* ^*407*^ (*Nipped-B* ^*407*^ */+*). *y w* (*y w/+* females and *y w*/Y males) were the controls for the *dm* ^*2*^ (*dm* ^*2*^/+ females) and *Tor* ^*ΔP*^ (*Tor* ^*ΔP*^/+) mutants. The data shown is from a single experiment and two additional independent repeats with *Nipped-B* ^*407*^ and P57B gave very similar results. Error bars are standard errors.

All genotypes showed a nearly 2-fold increase in the number of days to reach pupariation and eclosion and small insignificant decreases in viability when grown on 20% food (Fig 1). Under all conditions, heterozygous *Nipped-B* ^*407*^ mutant flies reached these stages at the same time as the matched wild-type parental control (P57B), with a similar number of surviving pupae and adults. In some experiments, female *Nipped-B* ^*407*^ mutants eclosed a few hours earlier than the P57B controls. Thus *Nipped-B* mutant flies do not show an altered rate of development or reduced viability, and the systemic growth controls are not detectably altered relative to wild-type when all tissues have the heterozygous *Nipped-B* ^*407*^ mutation.

As expected, *dm* mutants gave half the number of pupae as the matched *y w* control flies because hemizygous males do not survive. *dm* mutant heterozygous females, however, eclosed earlier than the *y w* control females on normal food, and almost two days earlier when grown under limited nutrition, demonstrating that heterozygosity for a null *dm* allele alters systemic growth control, permitting premature developmental transitions under restricted nutrition (Fig 1). *Tor* mutant females also eclosed slightly earlier than the *y w* controls under normal nutrition, but at the same time as controls under limited nutrition (Fig 1). Thus, *Nipped-B*, *dm*, and *Tor* mutants all behave differently with regards to developmental timing, depending on nutritional conditions. Critically, *Nipped-B* mutants did not show significant changes in the timing of transitions between developmental stages under either normal and limited nutrition, indicating that the reduced growth of *Nipped-B* mutants is not caused by inefficient utilization of nutrition, or changes in systemic controls that produce hormonally-induced transitions when a critical size has been reached.

### *Nipped-B* mutant wings have fewer and smaller cells

**Fig 2:**
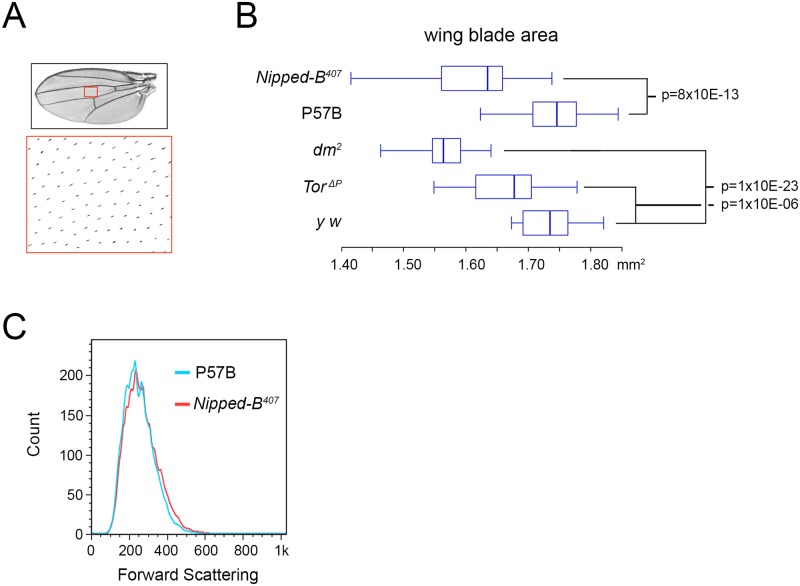
*Nipped-B*, *dm*, and *Tor* mutations reduce adult wing size. (A) Full adult female wing areas were measured, and areas of wings (red boxes) were sampled for the number of cells by counting the hairs produced by each cell at higher magnification (large red box) to determine cell size and number (Table 2). (B) Box plots showing the distributions of wing blade areas of the indicated genotypes: *Nipped-B* ^*407*^ (*Nipped-B* ^*407*^ */+*), P57B (P57B/+), *dm* ^*2*^ (*dm* ^*2*^ */+*), *Tor* ^*ΔP*^ (*Tor* ^*ΔP*^ */+*), *y w* (*y w/+*). The p values of various pairwise comparisons determined by the student t test are indicated. (C) Late 3^rd^ instar wing discs were dissected from larvae with the indicated genotypes, dissociated with trypsin and collagenase, and sorted by forward scattering in cell sorter to measure the distributions of cell sizes.

The three *Nipped-B* mutations tested (*Nipped-B* ^*407*^, *Nipped-B* ^*292*.*1*^, T(2;3)*Nipped-B* ^*359*.*1*^) dominantly reduce the size of adult wings to an extent similar to the reduction of body weight in *Nipped-B* ^*407*^ mutants (Table 2; S3 Fig). Examination of adult female wings indicates that *Nipped-B* ^*407*^ mutants are smaller because they have fewer and smaller cells. We measured the total wing area, and counted the number of cells per unit area using the hairs produced by each cell (Fig 2A and 2B; Table 2). From these data we calculated the total number of cells per wing, and the relative cell size (Table 2). Close analysis of the *Nipped-B* ^*407*^ mutant wings shows that the reduced size reflects a 4% decrease in cell number combined with a 3% decrease in cell size (Table 2). In Table 2, the increase in cells per mm^2^ from 5971 to 6163 measures the decrease in cell size. *dm* heterozygous mutant wings showed an approximately 9% reduction in size that reflects both a 4% decrease in cell number, and a 6% decrease in cell size (Fig 2B; Table 2). In contrast, *Tor* heterozygous mutant wings show only 3% decrease in wing area, with a decrease in cell size, but not cell number (Fig 2B; Table 2).

**Table 2:** Effects of heterozygous loss-of-function *Nipped-B*, *dm*, and *Tor* mutations on adult female wing size, cell size and cell number.

	Control		Mutant			Ratio of mutant to matched control		
	P57B	*y w*	*Nipped-B* ^*407*^	*dm* ^*2*^	*Tor* ^*ΔP*^	*Nipped-B* ^*407*^	*dm* ^*2*^	*Tor* ^*ΔP*^
wing area (mm^2^)	1.74	1.73	1.61	1.57	1.67	0.93	0.91	0.97
	± 0.01^a^	± 0.01	± 0.01	± 0.01	± 0.01			
			p<0.0001^b^	p<0.0001	p<0.0001			
cell area (μm^2^)	167.5^c^	161.9	162.3	153.2	156.0	0.97	0.95	0.96
	± 1.5	± 1.7	± 1.4	± 1.5	± 1.2			
			p = 0.01	p = 0.0002	p = 0.005			
cells per wing	10390^d^	10688	9922	10247	10706	0.96	0.96	1.00

^a^Standard error.

^b^P values determined using student t test.

^c^Calculated from cells per mm^2^.

^d^Calculated from the average wing area and cells per mm^2^.

We examined cell size in late 3^rd^ instar wing imaginal discs that give rise to the adult wings by forward scattering in a cell sorter after tissue dissociation, but did not detect a significant decrease in cell size at this earlier stage, when cells are still dividing (Fig 2C). It could be that the size difference is too small to detect by forward scattering, or that a significant difference in cell size occurs only after cells have stopped dividing.

### Ectopic *dm* expression restores wing size and increases the weight of *Nipped-B* mutants

**Fig 3:**
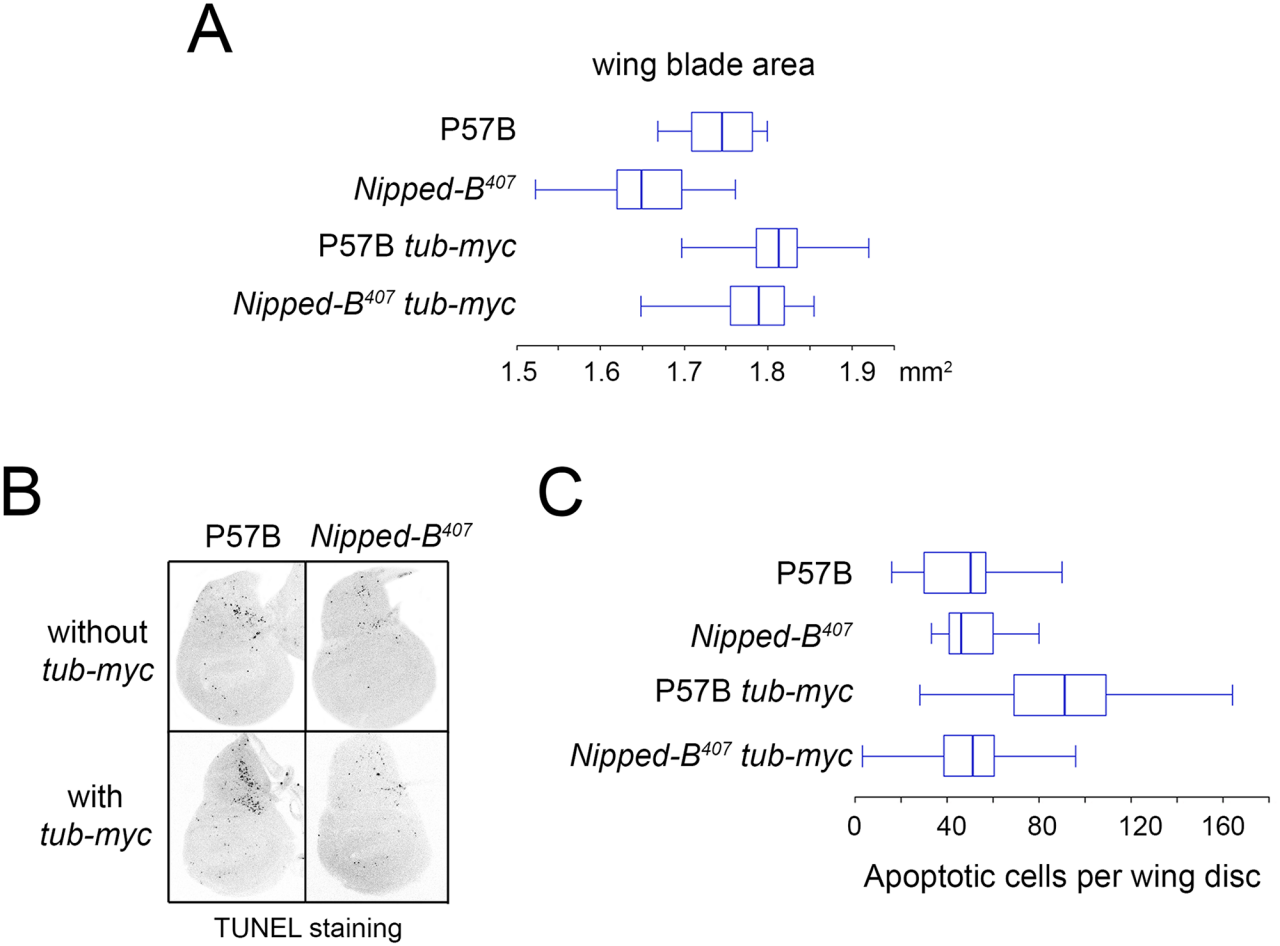
Additional *myc* (*dm*) expression rescues adult wing size of *Nipped-B* ^*407*^ heterozygous mutants and increases apoptosis in wild-type 3^rd^ instar wing imaginal discs. (A) The box plots indicate the distribution of the wing blade areas of the indicated genotypes, where *tub-myc* indicates a single copy of the *tub-myc* transgene that expresses Drosophila *dm* from a minimal tubulin gene promoter. The increases in wing area induced by *tub-myc* are significant for both the P57B / + and *Nipped-B* ^*407*^ / + genotypes (p<0.0001, t test). Cell numbers and sizes were also determined for each genotype and are given in Table 3. (B) Examples of TUNEL staining of late 3^rd^ instar wing imaginal discs of the indicated genotypes to visualize apoptotic cells. (C) Boxplot distributions of the number of apoptotic cells in 3^rd^ instar wing discs. The number of apoptotic cells in P57B / + discs with the *tub-myc* transgene is significantly higher than the other genotypes (p<0.0001, student t test).

Additional *dm* (*c-myc* ortholog) expression partially rescues the growth deficits of *Nipped-B* heterozygous null mutants (Fig 3A; Table 3). The Dm transcription factor increases protein synthesis, cell growth and cell proliferation [44]. Based on the knowledge that cohesin regulates *dm* expression and that *Nipped-B* mutant flies are small because of reduced cell number and size, we hypothesized that adding additional *dm* expression could rescue the growth of *Nipped-B* mutants. We used a *tubulin-dm* transgene (*tub-myc*, [45]) and observed an increase in the size of both *Nipped-B* mutant and parental wild-type control wings to a size greater than the normal wild-type (Fig 3A; Table 3). These results are consistent with the idea that the reduced growth of *Nipped-B* mutant flies may stem in part from reduced *dm* expression.

**Table 3:** Effects of additional *myc* (*dm*) expression on female wings and body weight.

	P57B Control	P57B *tub-myc*	*Nipped-B* ^*407*^	*Nipped-B* ^*407*^ *tub-myc*	Fold-change with *tub-myc*	
					P57B	*Nipped-B* ^*407*^
wing area (mm^2^)	1.74	1.8	1.65	1.78	1.03	1.08
	± 0.01^a^	± 0.01	± 0.01	± 0.01		
		p<0.0001^b^		p<0.0001		
cell area (μm^2^)	165.8^c^	175.7	160.9	171.9	1.06	1.07
	± 1.2	± 1.4	± 1.3	± 1.6		
		p<0.0001		p<0.0001		
cells per wing	11096^d^	10814	10260	10446	0.97	1.02
Adult body weight (mg)	1.37	1.33	1.26	1.33	0.97	1.03
	± 0.01	± 0.01	± 0.01	± 0.01		
		p = 0.01		p = 0.02		

^a^Standard error.

^b^P values determined using student t test.

^c^Calculated from cells per mm^2^.

^d^Calculated from the average wing area and cells per mm^2^.

Close examination of the wings showed that the increase in *Nipped-B* mutant wings caused by ectopic *dm* expression stems from both an increase in cell number and size, with an increase in the number of total cells and a decrease in the number of cells per mm^2^ (Table 3). In contrast, the wild-type control wings actually had fewer cells in the presence of the *tub-myc* transgene, and the increase in wing size thus results only from an increase in cell size (Table 3). Both mammalian *c-myc* and Drosophila *dm* promote apoptosis [46,47], and we thus tested if the *tub-myc* transgene reduces cell number in wild-type wings by increasing apoptosis. We quantified apoptotic cells in 3^rd^ instar wing imaginal discs by TUNEL staining and found that *tub-myc* increased the number of apoptotic cells some 1.7-fold in wild-type wing discs, but had no measurable effect in *Nipped-B* mutant discs (Fig 3B and 3C). In other words, the *Nipped-B* mutation suppressed apoptotic induction by excess *dm* expression. Importantly, these data indicate that *Nipped-B* mutants do not have increased apoptosis, even in the presence of excess *dm* expression, providing further evidence that they have fewer cells because of reduced cell proliferation, not increased cell death.

### Heterozygous *Nipped-B* mutant wing discs show broad modest decreases in expression of genes supporting development and growth

**Fig 4:**
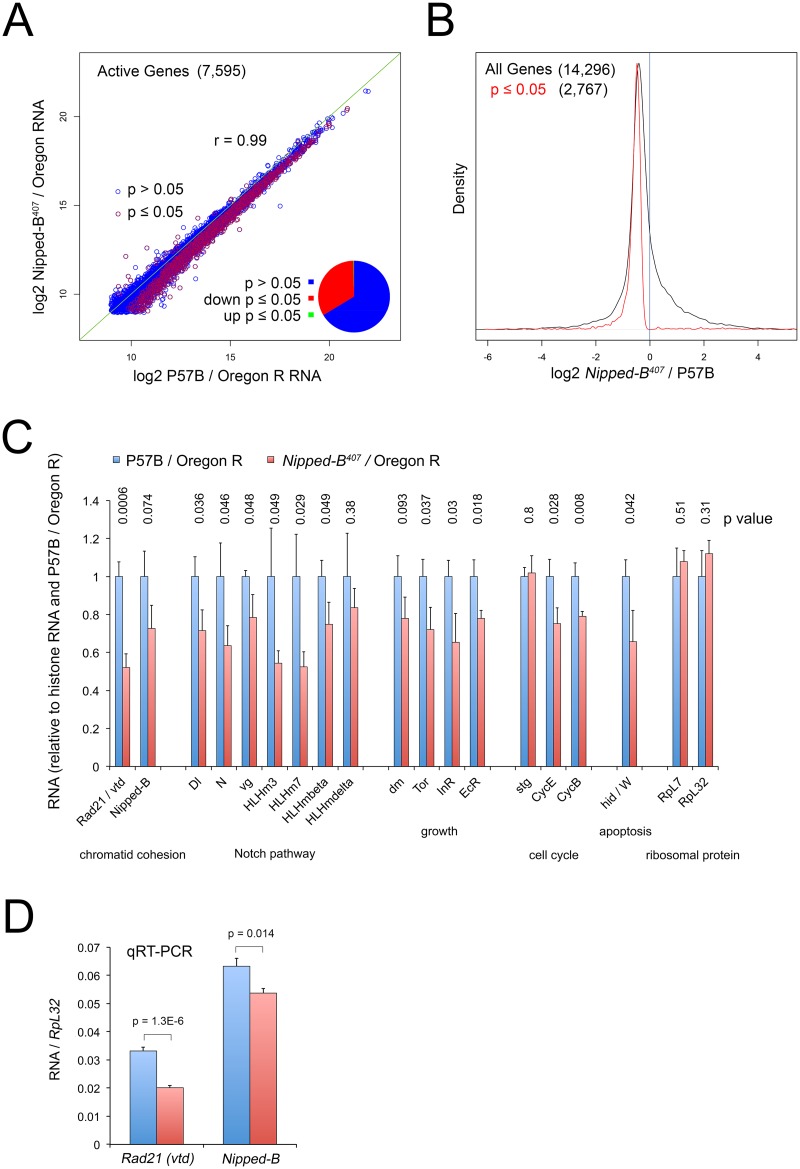
*Nipped-B* mutant wing discs show broadly reduced gene expression by RNA-seq analysis. (A) Plot of expression level of all active genes in matched wild-type controls (P57B / Oregon R) versus *Nipped-B* ^*407*^ / Oregon R 3^rd^ instar wing discs. RNA-seq was performed using ribosomal RNA depletion and normalization to histone RNA to compensate for the broad effects of Nipped-B on gene transcription [24]. Active genes (7,595) were defined as those averaging more than 500 nucleotides of sequence coverage between replicates. Pearson correlation coefficients between replicate samples for all genes quantified (14,296) ranged from 0.96 to 1.00, indicating high reproducibility (S1 Table). The green line indicates a slope of 1, revealing that the *Nipped-B* mutants show diminished expression of many genes. Red circles indicate genes in which the change in RNA levels have a p value ≤ 0.05, which represent roughly a third of active genes as shown by the pie chart. (B) Density distribution plots of the fold-changes in gene expression between *Nipped-B* mutants and the wild-type control (log2 *Nipped-B* ^*407*^ / P57B ratio) for all genes (black) and those genes for which the change has a p value ≤ 0.05. The number of genes in each group is indicated in parentheses. This shows that relative to histone RNA levels, most genes show lower RNA levels in the *Nipped-B* mutant wing discs, and that most statistically significant changes are decreases in expression. (C) Examples of expression changes for selected genes. The blue bars are set to 1 in the P57B / Oregon R control after normalization to histone RNA to allow all genes to be shown on the same scale. The error bars are the standard errors calculated from the four replicates, and p values are given above each gene. The *Rad21* (*vtd*) cohesin subunit gene and *Nipped-B* both show reductions. Several genes in the Notch signaling pathway show reductions, including the *Delta* (*Dl*) ligand, *Notch* (*N*), and direct Notch targets, including *vestigial* (*vg*) and genes in the *Enhancer of split* complex (*HLHm3*, *HLHm7*, *HLHmbeta*, *HLHmdelta*). Several genes that regulate growth also show reductions including *dm* (*myc*), *Tor*, the insulin receptor (*InR*) and the ecdysone hormone receptor (*EcR*). The *string* (*stg*) cdc25 cell cycle control gene does not show a reduction, in contrast to the *cyclin E* (*CycE*) gene that drives S phase and the *cyclin B* (*CycB*) G2 cyclin gene. The *Wrinkled* (*W*, *hid*, *head involution defective*) pro-apoptosis gene shows a significant decrease. The *RpL32* and *RpL7* ribosomal protein genes, which are often used as normalization controls for qRT-PCR experiments and Northern blots, do not show significant changes in expression. The expression values for all genes in the RNA-seq analysis are in S1 Table, along with the ontology analysis indicating that pathways involved in development, morphogenesis and growth are broadly affected, although the only significant phenotype in adult *Nipped-B* mutant wings is the reduced cell number and size. (D) qRT-PCR validation of the effects on *Rad21* (*vtd*) and *Nipped-B* RNA levels in 3^rd^ instar wing discs, normalizing to *RpL32* RNA levels. Ten P57B / Oregon R and eight *Nipped-B* ^*407*^ / Oregon R replicates were assayed. The error bars are standard error calculated from all PCR replicates, and the p values for the comparisons to the controls are indicated.

Beyond the decreased cell number and size, heterozygous *Nipped-B* mutant wing discs do not show obvious morphological defects in the absence of mutations in other genes. However, studies in cultured cells using acute RNAi depletion of Nipped-B or cohesin levels down to 10 to 20% of normal levels show that Nipped-B and cohesin directly regulate the transcription of many genes that control growth and development, such as *dm*, transcription factors and signaling proteins [24,35]. We thus performed RNA-seq analysis of *Nipped-B* ^*407*^ / Oregon R mutant and P57B / Oregon R 3^rd^ instar wing discs to see if we could identify changes in gene expression responsible for the reduced growth, and the suppression of *dm*-induced apoptosis. This revealed modest decreases in the expression of a large number of genes, including many that participate in the control of growth and development, but do not implicate a small set of candidate genes as the central cause of the growth deficit (Fig 4; S1 Table).

We used an alternative method for normalizing the RNA-seq data because prior studies using Nipped-B and cohesin depletion in cultured cells showed that standard normalization methods for genome-wide RNA analysis, which assume that most genes do not change in expression, obscure many small changes in gene expression. This is because Nipped-B and cohesin depletion alters the transcription of a majority of active genes via multiple direct and indirect mechanisms [24,35]. For instance, direct measurement of transcription by precision run-on sequencing (PRO-seq) showed that many genes with modestly reduced transcription showed increased RNA levels by standard normalization methods because their RNA decreases were smaller than the average decrease. Similar normalization artifacts have been encountered in genome-wide studies of the effects of the mammalian Myc protein on transcription because it amplifies transcription of most active genes [48]. We thus normalized to total histone RNA levels, reasoning that the tight regulation of histone mRNAs [49] will more accurately reflect DNA content and gene copy number. Histone mRNAs are not polyadenylated, and we therefore used ribosomal depletion instead of poly(A) selection to prepare the RNA-seq libraries.

With this approach, we find that roughly a third of active genes in *Nipped-B* mutant wing discs show modest but significant (p ≤ 0.05) decreases in RNA levels, with less than two dozen showing significant increases (Fig 4A and 4B; S1 Table). Active genes were defined as those that have at least 500 nucleotides of sequence coverage in the average of the replicate samples, which represents roughly half of all genes quantified, and captures genes that are expressed in only a few cells in the wing disc, such as *cut* and the *Enhancer of split* complex. The correlation in active gene expression levels between the averages of the P57B control and *Nipped-B* mutant samples is high (r = 0.99), and the correlation in expression for all genes between individual replicates ranged from 0.96 to 1.00, indicating the data is highly reproducible (Fig 4A; S1 Table).

*Nipped-B* RNA showed the expected decrease of 27%, which is only slightly greater than the median decrease in gene expression, and was not statistically significant (p = 0.074; Fig 4B and 4C; S1 Table). We confirmed that the decrease is significant using qRT-PCR of total RNA isolates with ten P57B and eight *Nipped-B* mutant replicates and *RpL32* as the normalization standard (p = 0.014; Fig 4D). We used *RpL32* as the reference because it did not show a significant change in the RNA-seq data (Fig 4C; S1 Table), and was also used as the standard in Northern blots showing a 25 to 30% decrease in *Nipped-B* mRNA in heterozygous *Nipped-B* ^*407*^ mutant larvae [6]. Combined with the sequence analysis showing that the bulk of *Nipped-B* RNA in *Nipped-B* ^*407*^ / Oregon R wing discs arises from the wild-type allele (S1 and S2 Figs) and the high resistance of *Nipped-B* RNA levels to *in vivo* RNAi [6], these results provide additional evidence for a homeostatic feedback mechanism that maintains *Nipped-B* mRNA levels.

Intriguingly, *vtd* (*Rad21*) cohesin subunit RNA levels were significantly reduced by some 50%, which is a larger decrease than the 27% reduction in *Nipped-B* (Fig 4C), which was also confirmed by qRT-PCR (Fig 4D). The reduced expression of both *Nipped-B* and this cohesin subunit may explain why heterozygous *Nipped-B* mutations generally have stronger phenotypic effects in Drosophila and humans than heterozygous cohesin subunit mutations.

Gene ontology analysis revealed enriched down-regulation of genes involved in development, morphogenesis and growth (S1 Table). These include known Nipped-B and cohesin targets in the Notch signaling pathway, including *Notch* (*N*) and genes in the *Enhancer of split* complex (*HLHm3*, *HLHm7*, *HLHmbeta*, *HLHmdelta*) (Fig 4, S1 Table). Although *dm* transcription in Nipped-B depleted cultured cells is substantially reduced as measured by PRO-seq [24], *dm* RNA is only modestly reduced by 22% in the heterozygous mutant wing discs, which did not achieve statistical significance (p = 0.09) (Fig 4C; S1 Table). Other growth regulators such as *Tor*, the insulin receptor (*InR*) and the ecdysone hormone receptor showed similar decreases with significant p values (Fig 4C, S1 Table). Consistent with the resistance of *Nipped-B* mutant wing discs to *dm*-induced apoptosis, the *Wrinkled* (*W*, *hid*) pro-apoptotic gene is also significantly reduced in expression (Fig 4C, S1 Table). The *CycE* and *CycB* genes controlling the cell cycle are also reduced, although the *string* (*Cdc25*) gene is not (Fig 4C; S1 Table).

Given the large number of modest reductions in genes involved in growth and development, the evidence for homeostatic mechanisms, and the inability to dissect direct from indirect effects, the RNA-seq data do not definitively identify a specific gene or set of genes responsible for the growth deficit of heterozygous *Nipped-B* mutant wing discs, and it is likely that many direct and indirect expression changes contribute to the reduced growth. We suspect that *dm* overexpression can partially rescue the growth deficit not only because it compensates for a modest direct decrease in *dm* transcription, but also because it broadly amplifies expression of many genes [48] and thus can counteract the broad down-regulation in gene expression caused by reduced Nipped-B.

### Heterozygous *Nipped-B* mutants show learning and memory deficits

Although heterozygous *Nipped-B* null mutants show no overt external structural abnormalities, we investigated whether or not they have deficits in neurological and behavioral functioning. Overall, intellectual disability is present in 95% of individuals with CdLS, and is often present even in the absence of significant structural abnormalities. Homozygous deficiencies of *SMC1*, *Stromalin*, and *Rad21* cause aberrant axon pruning of the Drosophila mushroom body γ-neurons, raising the possibility that there may be modest nervous system deficits in heterozygous mutants [50,51]. Mushroom bodies (MBs) are the primary brain structures involved in associative learning and memory, functions that can be tested through conditioning courtship paradigms and odor-shock classical conditioning paradigms [52–54].

We investigated the learning and memory capability of *Nipped-B* heterozygous males through courtship conditioning assays. Courtship is an innate behavior that involves a series of complex sensory and behavioral interactions between male and female *Drosophila*. Courtship rituals performed by the male fly is characterized by a sequence of stereotypic behaviors including: 1) orienting toward a female, 2) tapping the females abdomen with the males’ forelegs, 3) extending and vibrating one wing, 4) licking the female’s genitalia and 5) attempting to copulate [55,56]. Overall the courtship robustness of male flies is measured by the courtship index (CI), namely the proportion of time a male engages in active courtship behaviors during a given amount of observation time. Virgin females are receptive to copulation. Recently mated females, however, are non-receptive and reject advances to mate. The courtship activity of the male is modified by their prior sexual experience. Unsuccessful courtship attempts with a non-receptive female lead to progressive decrement of a male tester’s courtship behaviors. Such courtship suppression is retained even after the male is removed from the non-receptive female and later paired with a receptive virgin female [57]. Courtship plasticity observed in males is thus employed in conditioning courtship paradigms to measure learning and memory. During a training session, a naïve male is paired with pre-mated female “trainer” in a mating chamber and observed for 60 minutes. The amount of time the male engages in courting the trainer female is recorded for the first 10 minutes and the last 10 minutes and used to calculate the courtship indexes (CI), CI_initial_ (CI of the first 10 minutes) and CI_final_ (CI of the last 10 minutes), respectively. The discrepancy between CI_initial_ and CI_final_ thus reflects the extent of courtship reduction and a tester male’s capacity to learn. After the training session, the male tester is transferred into a new chamber and given an hour to rest. A receptive virgin female is subsequently introduced into the chamber. The courtship attempts of the tester male towards the virgin female are recorded for 10 minutes, which is used to deduce the courtship index CI_test_. A naïve fly of the same genotype is sham-trained in a courtship chamber for an hour in isolation and then tested for courtship activity towards a virgin female; the courtship of the naive fly is referred as CI_sham_. The difference between CI_test_ and CI_sham_ reflects the retention of courtship suppression resulting from the earlier unrewarding courtship experience and is used as a measure of short-term memory [58,59].

Two *Nipped-B* mutant alleles were used for the conditional courtship assays, *Nipped-B* ^*407*^ and *Nipped-B* ^*292*.*1*^. Like *Nipped-B* ^*407*^, *Nipped-B* ^*292*.*1*^ was one of the founder alleles defining the *Nipped-B* complementation group, and thus has also been extensively tested for a lack of mutations in the surrounding essential genes [5]. In the initial genetic characterization, *Nipped-B* ^*407*^ and *Nipped-B* ^*292*.*1*^ showed similar genetic interactions with other mutations on *cut*, *Ultrabithorax*, and *mastermind* mutations [5]. *Nipped-B* ^*292*.*1*^ also has a similar effect on adult female wing size as *Nipped-B* ^*407*^ (S3 Fig). To minimize potential influence of background variations on behavioral outputs, both *Nipped-B* mutants were backcrossed to an isogenized *w* ^1118^ strain, *w* ^*iso31*^, which are isogenic for chromosomes X, 2 and 3, and were rigorously tested to ensure that they have wild-type memory and circadian rhythm [60]. *w* ^*iso31*^ flies were used as controls in all *Drosophila* behavioral analyses in this study and heterozygous *Nipped-B* mutants were generated by crossing the isogenized *Nipped-B* ^*407*^ or *Nipped-B* ^*292*.*1*^ chromosomes into a *w* ^*iso31*^ background.

**Fig 5:**
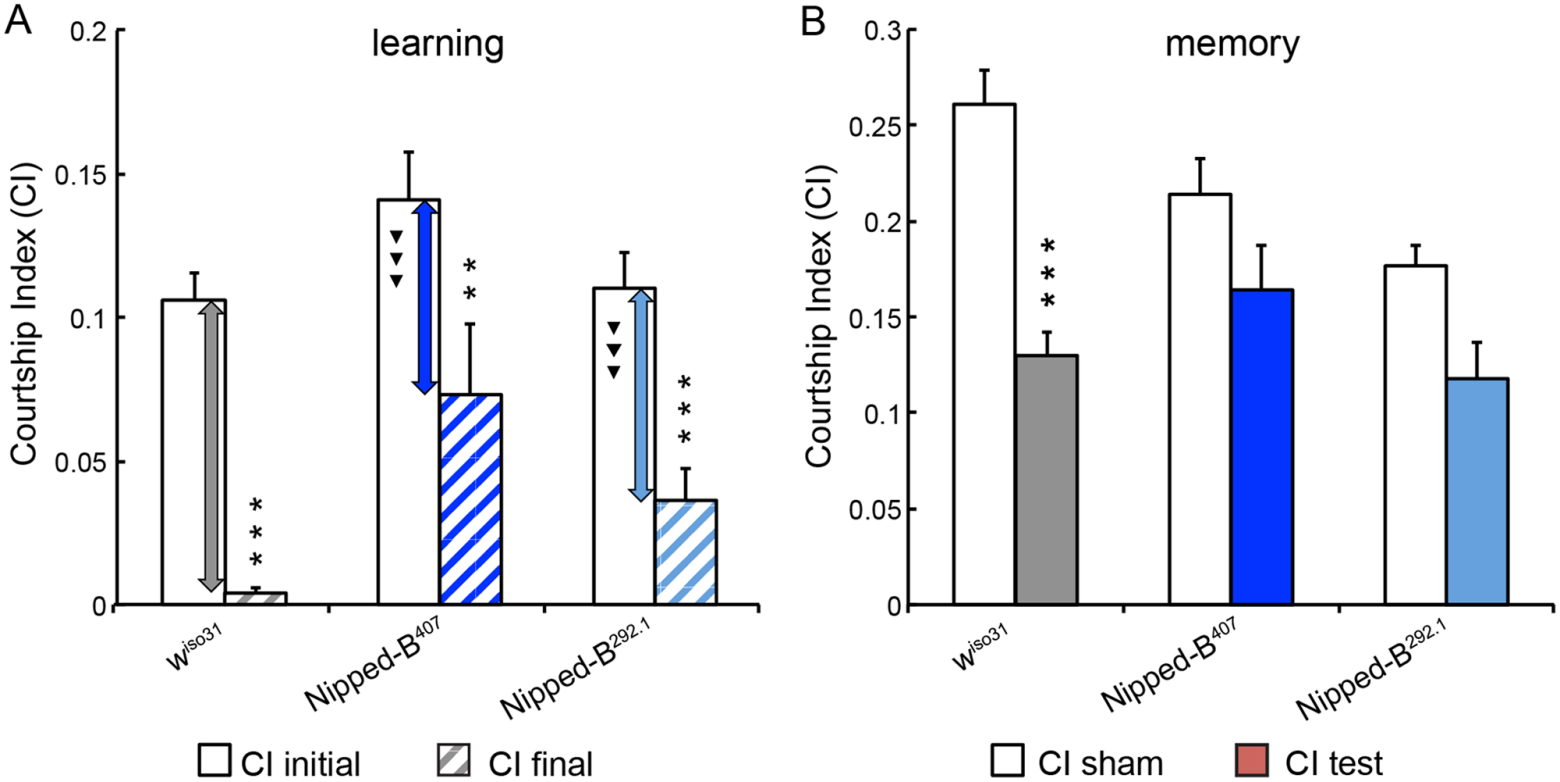
*Nipped-B* heterozygotes are deficient in learning and short-term memory. (A) Similar to *w* ^*iso31*^ controls (*p*<0.0001) heterozygous *Nipped-B* males of both alleles (*Nipped-B* ^*407*^ (*p* = 0.007) and *Nipped-B* ^*292*.*1*^ (*p*<0.0001)) show significant decrease in their courtship activities through training sessions with non-receptive pre-mated females, suggesting that these flies display learning in experience-based conditioning. However, when the amount of decline between CI initial and CI final is compared between each *Nipped-B* heterozygote and the *w* ^*iso31*^ controls, the decrements in the mutants are significantly smaller. (B) When tested for their courtship behaviors towards receptive virgin females after a short break (1 hour), trained *w* ^*iso31*^ controls show a near 50% reduction in their courtship behaviors (CI test) compared the naïve *w* ^*iso31*^ males (CI sham) (*p*<0.0001). However, heterozygotes of neither *Nipped-B* mutant show a significant difference to their naïve peers, for *Nipped-B* ^*407*^, *p* = 0.10 and for *Nipped-B* ^*292*.*1*^, *p* = 0.059. Over 18 flies were tested for each genotype in a given assay. Student’s t-tests were used for analyses with the levels of significance marked by number of *: *, *p*<0.05; **, *p*<0.01; ***, *p*<0.001. One-way ANOVA *post hoc* Tukey tests were used to decide statistical difference in learning capacity between *Nipped-B* ^*407*^ or *Nipped-B* ^*292*.*1*^ heterozygous males with *w* ^*iso31*^ controls; ^▼^, *p*<0.05; ^▼▼^, *p*<0.01; ^▼▼▼^, *p*<0.001.

We observed that similar to *w* ^*iso31*^ controls, heterozygous *Nipped-B* mutants of either allele demonstrated a substantial reduction in their courtship activities toward non-receptive female trainers, when their courtship behaviors during the first 10 minutes were compared to those seen in the final 10 minutes of a training session (Fig 5A). However, the reduction in courtship attempts seen in *Nipped-B* males was not as remarkable as that of *w* ^*iso31*^ controls. We compared the amount of courtship reduction in all three groups and noticed that heterozygous mutants of either *Nipped-B* allele show less decrease than *w* ^*iso31*^ controls. These results indicate that although heterozygous *Nipped-B* males (*Nipped-B* ^*407*^ / + and *Nipped-B* ^*292*.*1*^ / +) are capable of learning, their capacity is not as high as that of controls (Fig 5A). In addition, when tested with a receptive virgin female after an hour break, the conditioned heterozygous *Nipped-B* males (*Nipped-B* ^*407*^ / + and *Nipped-B* ^*292*.*1*^ / +) do not show significantly reduced courting attempts compared to the naïve males of the corresponding genotypes (Fig 5B, CI_sham_ vs. CI_test_). In contrast, age-matched *w* ^*iso31*^ controls did show significantly reduced courting attempts between the trained and naïve groups, which suggests that *Nipped-B* mutants have deficiencies in short term memory (Fig 5B).

### Heterozygous *Nipped-B* mutants have abnormal brain structures

Mushroom body morphology in *Nipped-B* heterozygous males was examined to see if underlying structural abnormalities might accompany the behavioral deficits. Mushroom bodies are not required for learning in courtship conditioning, but are essential for consolidation of short-term and long-term associative memories [52]. The adult Drosophila mushroom body is composed of three specific classes of neurons, α/β, α’/β’, and γ neurons. Their axon projections give rise to five terminal lobes, α, β, α’, β’, and γ lobes [57]. Although the exact roles of the specific neurons making up the mushroom bodies are still unclear, these discrete axon clusters can be distinguished by their distinctive projection patterns, differential expression of GFP driven by various Gal4 lines, and labeling with anti-Fasciclin II (anti-FasII) [51,61].

**Fig 6:**
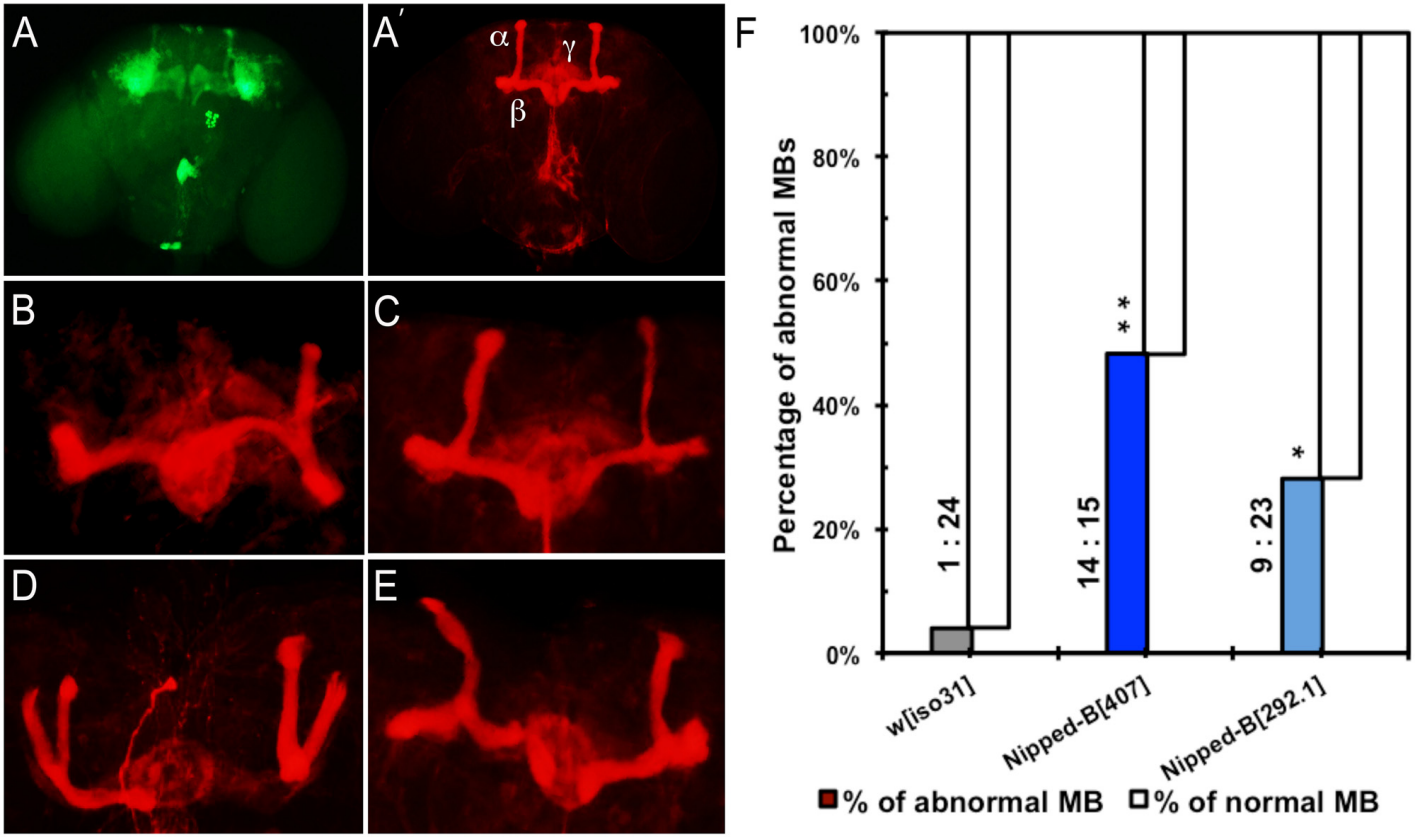
Mushroom body abnormalities seen in *Nipped-B* heterozygotes. Immunofluorescence images of 2 to 4 day old adult brains are shown. Mushroom bodies (MBs) are labeled by anti-FasII (shown in red) and *OK201Y>UAS-GFP* (shown in green). (A, A’) Wild-type mushroom bodies are shown. α, β and γ lobes can be distinguished by their distinct projection patterns and fluorescent labeling. *OK201Y >UAS-GFP* is expressed mainly in the γ lobes and weakly in the α and β lobes; anti-FasII labels the α and β lobes strongly and γ lobes weakly. (B-E) Abnormal MB morphology seen in *Nipped-B* heterozygotes. (B) short α lobe and β lobe missing. (C) thinner α lobe and β lobes. (D) β lobe missing. (E) kinky α lobe. (F) A higher percentage of *Nipped-B* heterozygous MBs are morphologically abnormal compared to controls. The differences are statistically significant. Numbers of abnormal MBs versus numbers of normal MBs counted in each genotype are labeled next to the columns. Statistical significance was determined through Fisher’s exact tests. *, p<0.05; **, p<0.01.

We observed that 48% of *Nipped-B* ^*407*^ heterozygotes (n = 29) and 27% in *Nipped-B* ^*292*.*1*^ heterozygotes (n = 32) display abnormal mushroom body morphology, compared to only 4% of controls (n = 25) (Fig 6). The mushroom body lobes (α, β, γ) in control and heterozygous *Nipped-B* mutant adult male flies were visualized by expressing an *OK201Y-Gal4>UAS-GFP* fluorescent reporter gene and immunostaining with anti-FasII antibody. The *OK201Y>UAS-GFP* reporter is strongly expressed in the γ lobe and weakly in the α and β lobes in adults, whereas anti-FasII strongly labels the α and β lobes and the γ lobes weakly. The increase in abnormal brain structures in both *Nipped-B* mutants is statistically significant relative to the control, although the difference between the two mutants is not.

The morphological changes in mushroom bodies of *Nipped-B* mutants are pleiotropic and generally affect only one side of the brain. Short or kinky α lobes, fused, detached, or missing β lobes (Fig 6B–6E) are the most common defects. We observed that among 29 *Nipped-B* ^*407*^ heterozygotes scored, 24% display missing or detached β lobe (n = 7), 10% show display short, kinky or detached α lobe (n = 3), and 7% show β lobe fusion with short or missing α lobe (n = 2). Among 32 *Nipped-B* ^*292*.*1*^ heterozygotes scored, the portion of flies of each above-mentioned category is 6% (n = 2), 9% (n = 3), and 3% (ne = 1), respectively. In addition, one of the 32 *Nipped-B* ^*292*.*1*^ heterozygotes displayed an α/β thinning phenotype. Although most of the structural abnormalities affect the α/β lobes, we also noted γ lobe defects in two *Nipped-B* ^*407*^ heterozygotes and one *Nipped-B* ^*292*.*1*^ heterozygote. In contrast to either *Nipped-B* mutant, only one out of 25 control flies showed a missing β lobe, with no other abnormalities detected. These morphological abnormalities demonstrate that heterozygous reduction of *Nipped-B* gene function interferes with brain development, and it is conceivable that these defects underlie the learning and memory deficits. Further studies will be required to substantiate this hypothesis and test the potential mechanistic connections.

### *Nipped-B* mutants are arrhythmic and sleep less than controls

Sleep problems are a common clinical finding in CdLS and other neurodevelopmental disorders. Disruptive sleep and/or circadian patterns have been observed in *Drosophila* models for Fragile X syndrome, Neurofibromatosis Type 1 and Angelman syndrome [62]. While the molecular and physiological function of sleep is still elusive, it has long been known to have a direct correlation to learning and memory. In fact, learning and memory deficits have been observed in all the aforementioned fly models. We investigated if *Nipped-B* heterozygotes, like the other fly models for neurodevelopmental disorders, have aberrant sleep and/or circadian rhythm patterns.

**Fig 7:**
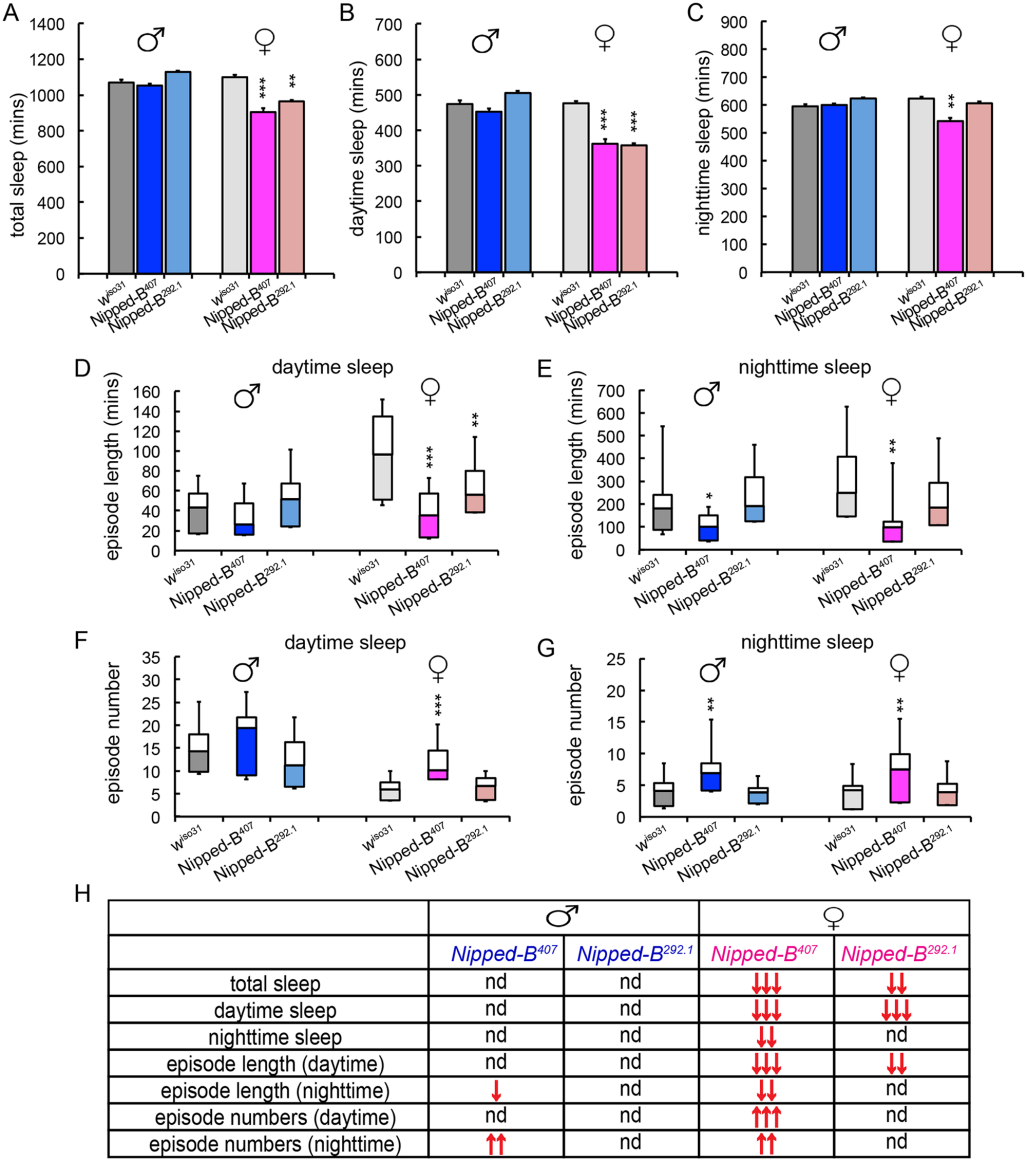
*Nipped-B* heterozygotes have disrupted sleep patterns. (A-C) *Nipped-B* heterozygous females, but not males, sleep less than *w* ^*iso31*^ flies as shown in amounts of total sleep (A), daytime sleep (B), and nighttime sleep (C). (D-G) In general, shortened sleep episodes seen in *Nipped-B* ^*407*^ male and female mutants are coupled with increased numbers of sleep episodes, both during daytime sleep and nighttime sleep. The lengths of sleep episodes of *Nipped-B* ^*292*.*1*^ females are not significantly different from *w* ^*iso31*^ flies. (H) Summary of statistical differences seen in *Nipped-B* mutants and *w* ^*iso31*^ controls. One-way ANOVA *post hoc* Tukey tests were used to determine statistical difference in the sleep parameters; *, *p*<0.05; **, *p*<0.01; ***, *p*<0.001. ↑ and ↓ indicate more or less/fewer than controls.

We measured the sleep patterns of *Nipped-B* heterozygous mutant flies by monitoring their locomotor activities under 12 hr light/12 hr dark cycles, which revealed significant sex-dependent alterations in sleep (Fig 7). One-way ANOVA Tukey *post hoc* tests were used to determine if the sleep parameters of the *Nipped-B* mutants differ significantly from those of *w* ^*iso31*^ controls. We found that both *Nipped-B* ^*407*^ and *Nipped-B* ^*292*.*1*^ heterozygous females sleep less than *w* ^*iso31*^ controls, in terms of both total sleep and daytime sleep (Fig 7A and 7B). In addition, the total amount of nighttime sleep of *Nipped-B* ^*407*^ heterozygous females was significantly less than *w* ^*iso31*^ controls (Fig 7C). Unexpectedly, there was no significant change in sleep totals in *Nipped-B* mutant males, suggesting that there are sexually dimorphic effects on the expression of genes that determine sleep patterns. While *Nipped-B* ^*407*^ mutant females showed reduced nighttime sleep, *Nipped-B* ^*292*.*1*^ females did not, indicating that daytime sleep is more sensitive to Nipped-B dosage (Fig 7D and 7E).

Compared to *w* ^*iso31*^ flies, the average lengths of sleep episodes were significantly shortened in *Nipped-B* ^*407*^ heterozygous females during both daytime and nighttime sleep (Fig 7D and 7E). Such reductions were partially compensated by an increased number of sleep episodes during the day and at night (Fig 7F and 7G). *Nipped-B* ^*292*.*1*^ females showed a significant reduction in sleep episode length during daytime sleep (Fig 7D), which was not accompanied by an increased number of sleep bouts (Fig 7F). The nighttime sleep episode length and numbers of *Nipped-B* ^*292*.*1*^ females were comparable to *w* ^*iso31*^ control females.

*Nipped-B* ^*407*^ and *Nipped-B* ^*292*.*1*^ heterozygous males sleep for similar amounts of time as *w* ^*iso31*^. However, a reduced sleep episode length during nighttime sleep coupled with a compensating increase in the number of episodes was also observed in *Nipped-B* ^*407*^ males (Fig 7E and 7G). All sleep parameters of *Nipped-B* ^*292*.*1*^ heterozygous males were not significantly different from *w* ^*iso31*^ controls. Fig 7H summarizes the complex patterns of sex- and allele-specific changes in sleep patterns.

**Fig 8:**
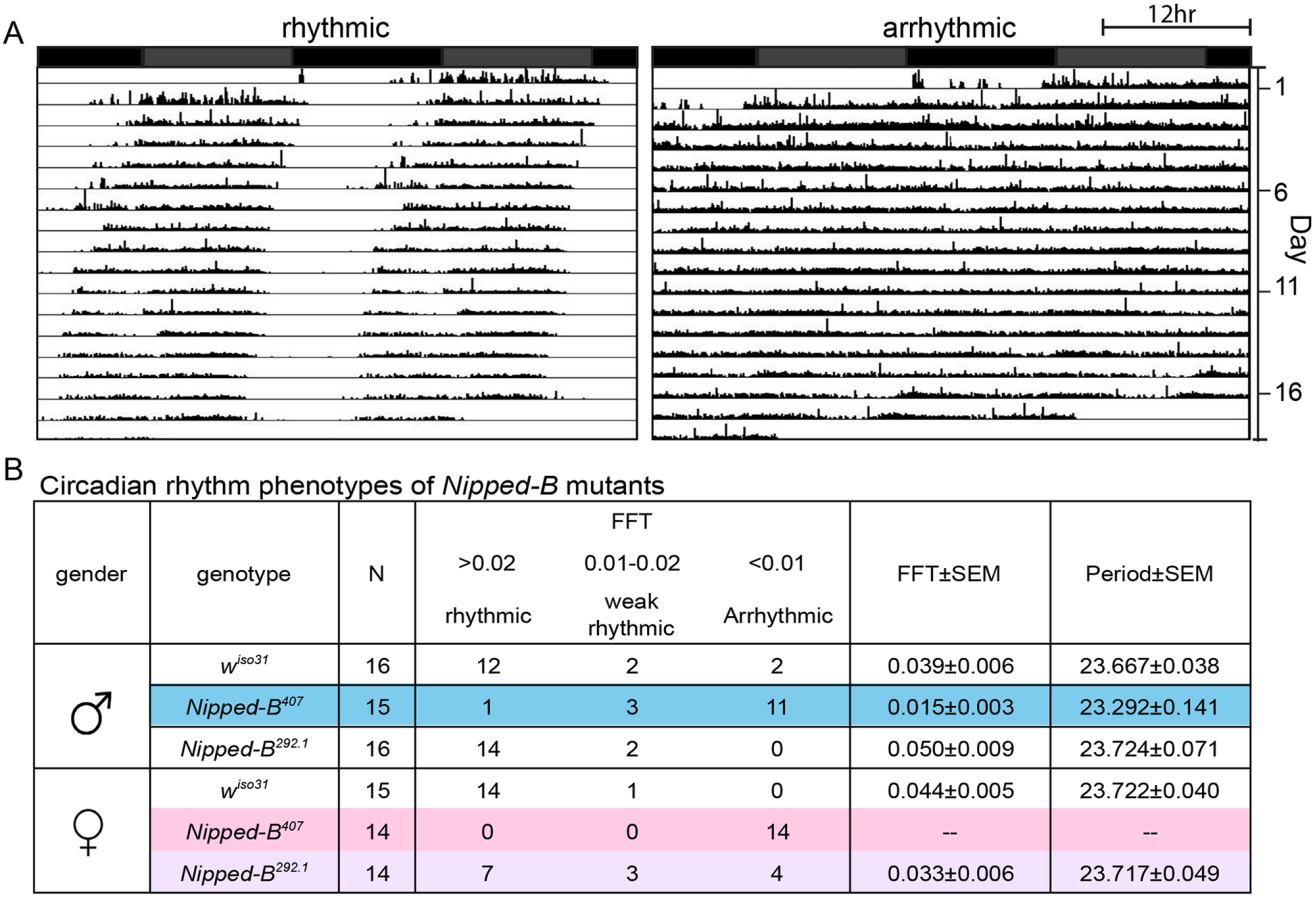
*Nipped-B* heterozygous mutants are arrhythmic. Flies were placed in constant darkness (DD) for over 14 days to record their locomotor activities, which were used to determine free-running rhythms. (A) Representative double plotted actograms are shown. Rhythmic flies show a robust rest/activity dichotomy, which is absent in arrhythmic flies. (B) Summary of circadian rhythm behaviors of *Nipped-B* mutants. Numbers of flies in each rhythmicity group are shown and N refers to the total numbers of flies tested. Rhythmicity was decided upon analysis of actogram, periodogram and FFT value. Significantly different from *w* ^*iso31*^ controls, most *Nipped-B* ^*407*^ males and all *Nipped-B* ^*407*^ females are arrhythmic (p = 0.001 for mutant versus control males shaded in blue, p = 0.001 for mutant versus control females shaded in pink, using Fisher’s exact test, low average FFT value). *Nipped-B* ^*292*.*1*^ females also show a slightly weaker rhythmicity than *w* ^*iso31*^ females (p = 0.042, Fisher's exact test, low average FFT value, shaded purple). FFT average and Periods were calculated from flies of the rhythmic and weak rhythmic groups. Periods of all genotypes were in the wild-type range (~23.5hr). SEM: standard error. FFT: fast Fourier transform value.

We investigated whether the aberrant sleep outputs seen in *Nipped-B* heterozygotes could arise from disruption of the circadian rhythm. Circadian rhythm is an intrinsic behavior independent of environmental cues. Four to five day old adult flies were loaded into a DAMS monitoring system to record their locomotor activity in constant darkness for over 14 days to measure their free-running circadian rhythms. The activity patterns of each fly were analyzed using the Clock program (Clocklab) to investigate periodogram of each individual fly to determine the period length and consolidation of the rhythm. In contrast to *w* ^*iso31*^ controls, a significant fraction of *Nipped-B* ^*407*^ males (p = 0.001, Fisher’s exact test) and all of the females (p = 0.0001) were arrhythmic (Fig 8). While the percentage of arrhythmic *Nipped-B* ^*292*.*1*^ heterozygous females is slightly higher than *w* ^*iso31*^ controls (*p* = 0.04, Fisher's exact test), there is no statistical difference between heterozygous males and the controls (*p* = 0.48) (Fig 8B). Periods calculated from rhythmic *Nipped-B* ^*407*^ males, *Nipped-B* ^*292*.*1*^ males and females, and *w* ^*iso31*^ controls were all within wild-type range (~23.5 hours) (Fig 8B). There is a clear correlation between circadian rhythm and sleep disturbances, with females being more strongly affected than males. However, *Nipped-B* ^*407*^ males show a strong circadian deficit, and only a modest change in sleep pattern, suggesting that circadian rhythm changes alone are insufficient to substantially alter sleep patterns.

Actograms averaging the activities of *w* ^*iso31*^, *Nipped-B* ^*407*^, and *Nipped-B* ^*292*.*1*^ males and females, and examples of additional individuals are presented in S4 Fig. Although the arrhythmic fly in Fig 8 appears to show more movement than the control, *Nipped-B* mutants are not hyperactive, as shown by the activity indices in S5 Fig.

### Heterozygous *Nipped-B* mutants show no signs of seizures

Seizure is another common neurological manifestation seen in CdLS individuals. We tested *Nipped-B* mutant flies to see if they are susceptible to seizures when subject to mechanical or thermal stress. Seizure-like behaviors in Drosophila induced by intensive vortexing are characterized by a sequence of initial seizure, temporary paralysis and recovery seizure [63]. We did not detect vortexing-induced seizures in *Nipped-B* mutant flies with either allele. We also tested these mutants in a heat-induced seizure assay [64] and *Nipped-B* mutants subject to brief heat-shock at 40° showed no sign of paralysis.

## Discussion

The studies reported here indicate that despite the large evolutionary divergence between Drosophila and humans, heterozygous *Nipped-B* mutant flies share both growth and neurological deficits with Cornelia de Lange syndrome. Although the mechanisms that cause these deficits in CdLS remain to be determined, the Drosophila studies suggest that at least some of the contributing mechanisms are highly conserved. The findings that Drosophila size is reduced primarily through mechanisms that do not involve systemic controls that sense body size in *Nipped-B* mutants, and that behavioral deficits are accompanied by morphological changes in brain structure are likely to have key implications for understanding the etiologies of the related deficits in CdLS.

### Role of Nipped-B in growth control

Prior studies revealed that *Nipped-B* heterozygous mutant *Drosophila* display subtle and latent external morphological phenotypes that become overt in adults only when combined with mutations in key developmental regulatory genes such as *cut*, *Ultrabithorax*, *Notch*, *mastermind*, *hedgehog*, and genes encoding cohesin or Polycomb silencing complex subunits [5,6,9,17,33–35]. This contrasts with mice and humans, where similar deficiencies in *NIPBL* cause multiple specific and obvious morphological changes [30]. There are also differences between mice and humans. For instance, limb abnormalities are largely absent in *Nipbl*(+/-) mice, but heart defects are significantly more frequent than in individuals with CdLS [14]. Extrapolating from Drosophila genetic interaction data, we posit that the individual physical birth defects in vertebrates stem from altered expression of specific sets of developmental genes, and that the variability between individuals with CdLS reflects differences in genetic background.

We show here that although the external morphological changes in *Nipped-B* mutant Drosophila are minimal in an otherwise wild-type background, they share the reduced size with *Nipbl*(+/-) mice and CdLS. The data argue that the reduced size reflects decreases in both total cell number and size, and not changes in the systemic control of growth that sense critical body mass, deficits in the utilization of nutrition, or increased cell death. The decrease in size likely stems in part from modestly reduced expression of the *myc* (*dm*), *Tor*, *InR* and other genes that promote cell proliferation, division and growth, and not increased apoptosis. Indeed, one of the most intriguing findings is the reduced ability of excess *dm* expression to induce apoptosis in *Nipped-B* heterozygous mutants. We speculate that this may stem in part from reduced function of a Dm-dependent enhancer that drives expression of the *grim* and *reaper* pro-apoptosis genes [65]. This region binds Nipped-B and cohesin in wing discs [35], and deletion of this region permits excess *dm* expression using the *tub-myc* driver to increase wing size more than in wild-type flies because it reduced apoptosis [65].

It remains to be seen to what extent these findings in *Drosophila* might explain the reduced growth in CdLS and in *Nipbl*(+/-) mice. It seems likely that similar mechanisms at least make a significant contribution to the reduced growth in mammals because *Nipbl*(+/-) mice and cells from individuals with CdLS also show reduced *c-myc* expression [14,15]. Organisms use a variety of mechanisms to sense body size and regulate growth and developmental transitions. Drosophila transitions are timed by pulses of the ecdysone steroid hormone produced by the prothoracic gland located between the two lobes of the brain [43]. Specific neurons in the brain secrete a peptide hormone, prothoracicotropic hormone (PTTH) that stimulates ecdysone production. Insulin signaling, nutrition and many other factors, which are not all well understood, control the hormonal pathways and timing of the ecdysone pulses that determine absolute body size. For example, nutrient deprivation substantially delays the ecdysone pulses, but not enough to fully restore maximal body size. What is particularly striking is how close *Nipped-B* mutants are to wild type in their developmental timing and in how their developmental staging responds to nutrient deprivation. This argues that the systemic hormonal pathways that regulate body size and hormonal pulses that induce developmental stages are largely unaffected, and that the reduced size of *Nipped-B* mutants stems primarily from a small but significant reduction in the number of cell divisions, and in the mechanisms that determine final cell size.

It is more difficult to precisely time the developmental staging in mice and humans than in *Drosophila*, and thus to determine whether or not developmental timing or systemic growth pathways are significantly altered with decreased NIPBL function. Although some individuals with CdLS show slightly delayed puberty, puberty occurs at the normal age in many [30]. The slight delays, and incomplete pubertal changes might all be attributed to causes other than a general developmental delay, such as structural abnormalities or changes in hormone levels. The relatively normal timing of puberty, therefore seems to indicates that the more extreme reductions in overall size observed in CdLS are also more likely to stem from changes in cell number and size than changes in systemic body size controls.

### Role of Nipped-B in neurological function

We also find that *Drosophila Nipped-B* heterozygotes display many behavioral and neurological features resembling those seen in CdLS patients: they are deficient in learning and short-term memory, and display disruptive sleep patterns and abnormal circadian rhythms. Intellectual disability is the most common clinical phenotype seen in individuals with CdLS. The average IQ score of typical CdLS cases, mostly with *NIPBL* mutations, is 53 (range 30–86) [32]. We find that although *Nipped-B* heterozygous mutant flies are capable of learning, their learning capacity is significantly lower than that of the *w* ^*iso31*^ controls. Furthermore, these *Nipped-B* mutants are accompanied by pleiotropic structural abnormalities in mushroom bodies, the major brain structures controlling learning and memory. While it is conceivable that the morphological defects in the mushroom bodies could contribute to the learning and memory deficits observed in the *Nipped-B* mutants, how these structural and functional deficiencies correlate with each other warrant further detailed studies.

We observed a striking similarity in the sleep patterns of fly *Nipped-B* heterozygotes and those seen in CdLS patients. Sleep disturbances are common in CdLS and seen in up to 55% of CdLS individuals. Insomnia (difficulty in initiating sleep), difficulty staying asleep, frequent night wakenings, and sleepiness during the daytime are the most common sleep problems reported in CdLS [66,67]. Frequent but dramatically shortened sleep episodes was the characteristic sleep pattern observed in *Nipped-B* heterozygote flies. In fact, the reduced length of sleep episodes lead to a dramatic loss in daytime and nighttime sleep, which was unable to be compensated for by significant increases in the number of sleep bouts.

While it has been speculated that sleep disturbances in CdLS individuals may be in part attributable to a circadian rhythm disorder, strict and objective studies to confirm this suggestion have not been undertaken. The locomotor activity-based circadian rhythm assay is a well-established measure of circadian rhythm in Drosophila. We demonstrated that an aberrant circadian rhythm exists in a large fraction of *Nipped-B* haploinsufficient male and female flies. For *Nipped-B* mutants that still display rhythmic circadian patterns, their free-running activity rhythms are maintained at around 24 hours, similar to the periodicity of *w* ^*iso31*^ controls. A remarkable consistency between the circadian defects and sleep aberrance was observed in these *Nipped-B* heterozygous mutants; flies that were profoundly arrhythmic were the ones that showed the most disturbed sleep patterns. Nevertheless, the circadian rhythm alterations are more apparent than the sleep disturbances in males, suggesting that additional sex-specific factors are involved in determining the sleep patterns. Taken together, these data suggested that at least in fly *Nipped-B* mutants, intrinsic circadian rhythm defects likely contribute to their aberrant sleep patterns.

Overall, our studies on fly *Nipped-B* mutants demonstrate a strikingly analogous growth and neurocognitive/behavioral phenotype between heterozygous *Nipped-B* mutants and human CdLS individuals, including small body size, learning and memory deficits, disruptive sleep patterns and circadian rhythm defects. Drosophila *Nipped-B* heterozygotes are thus a valuable resource, with multiple objective and readily measureable metrics, for modeling human CdLS. Studies on the function of *Nipped-B* and cohesin components in CdLS patient cell lines, *Nipbl(+/-)* mouse and zebrafish models, and Drosophila have contributed substantially to our understanding of the roles of *Nipped-B* and cohesin components in development and gene regulation. The presence of a sophisticated genetic tool kit and economical availability of fruit flies will make it possible to explore developmental deficits in a tissue- and stage-specific manner, as well as to test their relevance to human development and the pathogenesis of CdLS. Drosophila is an ideal model organism to address these issues, given its short life cycle, lower degree of genomic redundancy and the available genetic tools. *Nipped-B* mutants can also be utilized to search for and test new pharmacologic therapeutic modalities, towards amelioration of the growth and neurodevelopmental functioning of individuals with CdLS.

## Materials and Methods

### Drosophila culture

For growth and nutrition studies, flies were cultured on cornmeal, yeast and molasses media in agar [68] at 25°. To limit nutrition (20% food), the normal food recipe was diluted 5 fold in 0.9% agar in phosphate buffered saline (PBS). For all experiments, the mutant and controls were grown at the same time with the same batch of food preparation. To minimize differences in the genetic backgrounds, females with the balanced mutations or matched controls [(*y w*; *Nipped-B* ^*407*^ P{w+}57B/CyO, Kr-GFP), (*y w*; P{mw+}57B), (*y w*, *y w dm* ^*2*^/FM7), (*y w*; *Tor* ^*ΔP*^ P{neoFRT}40A/CyO, Kr-GFP)] were outcrossed to wild-type Oregon R males for all experiments, generating GFP negative progeny that are heteroallelic across the genome. Control and mutant crosses were always performed at the same time, and used the same batch of food.

*Nipped-B* ^*407*^ was chosen for detailed analysis in growth experiments because it has been shown to be rescued by *Nipped-B* cDNA transgenes, providing evidence that *Nipped-B* ^*407*^ is the only essential gene mutation in the chromosome [36,42]. The different fusion cDNA transgenes (FLAG-Nipped-B, Nipped-B-EGFP) used a full length cDNA of one alternatively-spliced *Nipped-B* transcript and were expressed using the constitutive *Chi* gene promoter. Depending on the transgene, they rescued Nipped-B^*407*^ homozygous mutants far beyond the normal 2^nd^ instar lethal phase to pharate adults, or viable fertile adults. Even with the most complete rescue, however, the flies showed reduced viability and were difficult to maintain, indicating incomplete rescue. The incomplete rescue likely reflects the lack of normal *Nipped-B* promoter regulation and splicing patterns.

To minimize potential influence of background variations on behavioral outputs, both *Nipped-B* alleles were backcrossed to an isogenized *w* ^*1118*^ strain (*w* ^*iso31*^), which was used as the control in all *Drosophila* behavioral analyses. For all the behavioral assays, heterozygous *Nipped-B* mutants were generated by crossing the isogenized *Nipped-B* ^*407*^ or *Nipped-B* ^*292*.*1*^ chromosomes into *w* ^*iso31*^ background and compared to the *w* ^*iso31*^ controls.

### Genomic DNA sequencing

Genomic DNA was purified from ~100 homozygous *Nipped-B* ^*407*^ mutant 2^nd^ instar larvae using the Zymo Research Fungal/Bacterial DNA MicroPrep kit according the manufacturer’s instructions and 50 nanograms of purified DNA was used to prepare a sequencing library with the Ion Xpress Plus Fragment Library Kit (Life Technologies) following the supplier’s methods with the exception that final library size selection was performed using Beckman Agencourt AMPure beads. The library was sequenced with an Ion Torrent Proton (Life Technologies) with a mean read length of 175 bp and aligned genome coverage of ~25-fold. Alignment to the Drosophila release 5 genome sequence (April 2006, modified by removal of chrU and Uextra sequences) was performed using the TMAP aligner map4 algorithm [69] with no soft-clipping and a minimum seed length of 20. The resulting bam files were sorted and indexed using SAMtools [70]. The sequence of the *Nipped-B* gene and surrounding region was inspected for indels and nucleotide changes in the aligned sequence files with the Affymetrix Integrated Genome Browser (IGB) [71].

### Determination of body weight

Adult flies were aged for two to three days after eclosion at weighed in batches of 20. Four to fourteen batches (80 to 280 total flies) were weighed for each genotype.

### Wing size, cell number and cell size measurements

Wings were dissected and mounted on glass slides under coverslips as previously described [72]. Photographs were taken at low magnification (4X objective) to quantify the wing area using ImageJ, and higher magnification (60X objective) of the same smaller region in each wing to count the number of cells per unit area. Cell number was counted by the number of wing hairs present in the region using a semi-automated method. The contrast of the digital high magnification photograph was increased to permit automated counting with the colony counting feature of AlphaEase software (AlphaInnotech). At least forty wings were measured per genotype. To measure cell size in developing wings, twenty wing disks each from male late 3^rd^ instar *y w*; *Nipped-B* ^*407*^ P{w+}57B/+ and *y w*; P{w+}57B/+ were incubated with collagenase (2 mg per ml) for 25 min in Ringer’s solution, transferred into 0.3 ml of Schneider’s medium containing 0.5% trypsin for 2.5 hours with periodic pipetting to dissociate the cells, and analyzed by forward scattering in a FACS.

### Developmental timing

Forty to fifty mutant or control virgin females were crossed to a similar number of Oregon R males in small beakers with apple juice plates covered with fresh yeast paste. Embryos were collected for 3 to 4 hours, and newly hatched larvae lacking the GFP balancers were collected 23 to 26 hours after the start of egg-laying and distributed into food vials (100 or 20% food) at 100 larvae per vial. Five to ten vials (500–1000 total larvae) were scored daily for pupariation and eclosion for each genotype. The experiments for *Nipped-B* ^*407*^ and the matched control were repeated three times. To examine the timing of the 2^nd^ to 3^rd^ instar molt, egg laying was restricted to a two hour period, and newly-hatched larvae collected was restricted to a one hour window.

### TUNEL staining

Late third instar wing discs from *y w*; *tub-myc*/P57B, *y w*; *tub-myc*/*Nipped-B* ^*407*^ P57B, *y w*; +/P57B, and *y w*; +/*Nipped-B* ^*407*^ P57B were fixed in 4% paraformaldehyde in PBS and stained using the Click-iT TUNEL Alexa Fluor 488 Imaging Assay (Molecular Probes) according the manufacturer’s protocol. The stained discs were imaged using a Leica SP5 laser scanning confocal microscope to count the number of apoptotic cells. Twenty discs were scored for each genotype, and the experiment was repeated three times.

### RNA-seq expression analysis

Total RNA was isolated from late 3^rd^ instar wing discs of heterozygous P57B control and *Nipped-B* ^*407*^ mutant female larvae after crossing to Oregon R wild type, in three separate collections from independent crosses using Zymo Research Quick-RNA MicroPrep Kits with DNaseI treatment following the manufacturer’s protocol. Ribosomal RNA depletion and sequencing library construction for each wing disc RNA isolate were performed using Eukaryotic RiboMinus and Ion Total RNA-seq v2 kits (Life Technologies) according to the manufacturer’s directions. Sequencing was performed on an Ion Torrent Proton with a mean read length of 135 nucleotides, and reads were aligned to the release 5 Drosophila genome (April 2006) using the TMAP aligner map4 algorithm. Soft-clipping at both 5’ and 3’ ends of the reads was permitted during alignment to accommodate spliced reads, with a minimum seed length of 20 nt. One P57B and one *Nipped-B* ^*407*^ library were sequenced twice to provide additional replication, and were used as fourth replicates for statistical calculations. The combined coverage of sequencing for each genotype was >80-fold genome equivalents, with roughly half the reads corresponding to ribosomal RNA that was incompletely removed by depletion.

RNA-seq analysis methods typically normalize to total reads, which assumes that most genes do not change in expression under experimental conditions, but this assumption is false when Nipped-B or cohesin levels are reduced [24]. We thus normalized to total histone RNA, which is tightly controlled to reflect genomic DNA content [49]. To accommodate this normalization, the TMAP aligner parameters were set such that each histone RNA read was assigned randomly to only one of the top matches, and the U and Uextra sequences were removed from the genome sequence to prevent assignment of reads to unannotated histone gene copies. Genome-wide strand-specific nucleotide coverages were calculated from the aligned bam files for each sample using the *genomecoveragebed* program in BEDTools [73] and the nucleotide coverage for all non-redundant exons for each gene were summed using custom R [74] scripts. Most RNA-seq methods calculate expression using total read numbers, which assumes that the reads are the same length, but calculating total nucleotide coverage is more accurate for Ion Torrent sequencing, which produces reads of differing lengths. After removal of the contaminating ribosomal RNA coverage, and the coverage of small non-coding RNAs (snoRNAs, tRNAs, miRs, etc) which are not quantitatively removed during the library size selection step, the total histone RNA coverage for each sample was calculated and used to normalize the nucleotide coverage for all other genes in each sample. Normalization factors were calculated by averaging the total histone coverage for all replicates and dividing this average by the histone coverage for each sample. The total coverage for each gene in each replicate was then multiplied by these factors after adding an offset of 1 to each gene to preclude division by 0 in subsequent calculations. The averages, standard errors and p values of the coverage values for all genes in the four P57B and Nipped-B replicates (three independent RNA isolations and one resequencing for each genotype) were calculated using Microsoft Office Excel, and additional analysis including calculating correlation coefficients was performed using R. The data is presented in S1 Table and deposited in the GEO database, accession no. GSE74192. The expression values in S1 Table are the normalized strand-specific nucleotide coverage for each gene.

### Courtship training and testing

Male and female flies used for courtship testing were prepared as described in [58] and [75]. Briefly, males of the appropriate genotype were collected within 4 hours of eclosion and kept individually in small food-containing vials. Virgin *X^X*, *y f* females were collected in the same manner and kept in groups of 10 to 15 in standard food vials. All flies were aged at 25° in a 12:12 LD cycle and courtship testing was performed during the relative light phase. Trainer females were 5 days old on the day of testing and were copulated the day before testing. Virgin females used on the day of testing were 5 days old. Male tester flies were 5 days old and were randomly assigned into training group and naive groups. The experimenter was blinded to the genotypes of the males used in courtship testing. The total amount of time a tester male engaged in active courting (orienting, taping, singing, licking and attempting copulation) was recorded for a period of 10 minutes, or upon successful copulation. The courtship index (CI) is defined as the percentage of time spent in courting during a 10 minute period or before successful copulation. For each genotype, at least 18 males were tested in the naive group and the training group. Statistical analyses were performed as described in [58].

### Sleep and circadian rhythm assays

Sleep and circadian rhythm tests were performed and analyzed as described [76,77]. Adult flies were kept as groups of males and females and entrained to a 12hr light: 12hr dark (L/D) cycle at 25°C for 3 days prior to testing. They were then individually transferred into glass tubes and monitored through the Drosophila Activity Monitoring System (Trikinetics, Waltham, MA). A beam of infrared light passes through the middle of the tube and fly activities were deduced from beam-breaking events. Behavioral quiescence longer than 5 minutes is defined as sleep. Sixteen flies of each sex were tested for each genotype. For sleep assays, the flies were loaded into the monitors at 9 am and then monitored for 6 days in L/D cycle. Sleep data collected were processed with Pysolo software [78] to calculate the amount of sleep, the number of sleep bouts, and sleep duration. For circadian assays, the flies were loaded into the monitors at 9 pm and data were collected from flies monitored in constant darkness for at least 14 days. Data collected were processed with ClockLab (Actimetrics, Wilmette, IL) to generate periodograms and actograms. Period length and FFT value (Fast Fourier Transformation, the relative power of rhythmicity) were collected from periodogram. Rhythmicity is decided by reviewing both actogram and periodogram. Statistical analyses were performed as described in [77,79].

### Mushroom body labeling and morphological analysis

Brains from 2–4 day old adults were dissected in PBS, fixed, and stained as described [59,76]. Anti-Fasciclin II antibody (1:10 dilution, ID4, Developmental Studies Hybridoma Bank, IA) and Alexa-568 goat anti-mouse secondary antibody (1:400 dilution, Life Technologies, Carlsbad, CA) were used. Tissues were mounted in VectaShield (Vector Laboratories, Burlingame, CA). Images were acquired with an Olympus 1X71 fluorescence microscope. For each genotype, at least 18 males were tested in the naive group and in the training group. The experimenter was blinded to the genotypes of the brains using in morphology analyses.

### Seizure tests

Mechanical stress tests (bang sensitivity) and heat shock assays were performed as described [63,80]. Over 50 flies were tested fro each stress test.

## Supporting Information

S1 Fig The bulk of *Nipped-B* RNA in *Nipped-B* ^*407*^ / Oregon R wing discs is from the wild-type allele. The top diagram shows the location of *Nipped-B* in centromere-proximal heterochromatin on chromosome 2R, with extensive repeat sequences, including many natural transposable elements (TEs, pink and purple). The *Nipped-B* gene displays histone H3 lysine 9 trimethylation (H3K9me3) and HP1 occupancy typically associated with heterochromatin. The homozygous *Nipped-B* ^*407*^ genomic sequence shows that nucleotide 579,589 (release 5 April 2006 coordinates) in the second to last exon matches the reference genome (T in plus strand, A in minus strand), while the RNA sequence from *Nipped-B* ^*407*^ / Oregon R wing discs does not, with all aligned reads showing C in the minus strand (G in plus strand). This is a silent mutation in the wobble position of a Thr codon that does not change the amino acid sequence. Thus most of the RNA is not produced by *Nipped-B* ^*407*^, suggesting that is essentially a null allele. No mutations were found in the *Nipped-B* ^*407*^ coding sequences by genomic sequencing. RNA-seq sequence coverage shown in S2 Fig further indicates that there are no alterations in transcription start and termination, or in splicing. Thus *Nipped-B* ^*407*^ is likely a chromosomal rearrangement (insertion, deletion, transposition or inversion) that is hidden by the repetitive sequence nature of the surrounding environment.
(TIF)

S2 Fig The *Nipped-B* ^*407*^ mutation does not cause new transcription start sites, new termination sites, or alter splicing in wing discs. The top shows the sequence coverage (log2 bedgraph) for the minus strand genome sequence of *Nipped-B* ^*407*^ homozygous 2^nd^ instar larvae (orange), and for the minus strand RNA-seq coverage for P57B / Oregon R wing discs (black) and *Nipped-B* ^*407*^ / Oregon R wing discs (blue). The mathematical difference between the P57B and *Nipped-B* ^*407*^ log2 RNA-seq coverage (ΔP57B-*Nipped-B* ^*407*^) is shown in the bottom track (green) to visually compare the transcript patterns in P57B and *Nipped-B* ^*407*^. This subtraction, which calculates fold-differences, reveals that there are no significant changes in coverage across the gene between the P57B control and *Nipped-B* mutant wing discs, indicating that there are no changes in the transcription start sites, termination sites or splicing. The middle panel shows the 5’ end of the gene, where there is substantial alternative splicing, to show that the splicing pattern is not detectably altered. The bottom panel shows the log2 RNA-seq bedgraphs (combined plus and minus strand transcription) for the entire region surrounding *Nipped-B*, indicating that transcription of the genes neighboring *Nipped-B* are not altered by the *Nipped-B* ^*407*^ mutation. Combined with the sequence comparison in S1 Fig, this indicates that the bulk of *Nipped-B* RNA in *Nipped-B* ^*407*^ / Oregon R wing discs is from the wild-type allele.
(TIF)

S3 Fig Heterozygous *Nipped-B* ^*292*.*1*^ and T(2:3)*Nipped-B* ^*359*.*1*^ alleles reduce adult female wing size in Oregon R and Canton S heteroallelic backgrounds. The top panel is a boxplot of the wing blade areas of females of the indicated genotypes, and the bottom panel gives the p values for the indicated comparisons (t test).
(TIF)

S4 Fig Actograms of *Nipped-B* heterozygous mutants and *w* ^*iso31*^ control flies. (A1-C1, A2-C2) Average double plotted actograms for *w* ^*iso31*^, *Nipped-B* ^*291*.*1*^, and *Nipped-B* ^*407*^ flies. The numbers of flies in each genotype are described in Fig 8B. The mutants that are significantly different from controls in rhythmicity are denoted by *. (A3-C3, A4-C4) Representative double plotted actograms of individual flies of the indicated genotypes.
(TIF)

S5 Fig Activity and sleep profiles of Nipped-B heterozygous mutants and wiso31 control flies. (A1, B1) Average activity patterns of *Nipped-B* mutant and control males (A1) and females (B1) recorded in 12 hour L/D cycles (15 to 16 flies of each genotype and sex for over six days). The activity of both male and female *Nipped-B* mutants is comparable to that of wild-type controls, suggesting that they are not hyperactive. (A2, B2) Sleep profiles for males (A2) and females (B2) with standard error bars. These patterns were analyzed in more detail in Fig 7.
(TIF)

S1 Table RNA-seq analysis of gene expression in *Nipped-B* ^*407*^ / Oregon R and P57B / Oregon 3^rd^ instar wing discs. The spreadsheet tabs give the expression values for active genes (> 500 nt average normalized coverage) (EXPRESSED), the active genes that decrease in expression with a p value ≤ 0.05 (DOWN), the functional gene ontology analysis [81,82] of the active genes that show a decrease in expression, the active genes that show an increase in expression with a p value ≤ 0.05 (UP), the Pearson correlation coefficients between all sample replicates (REPLICATE CORRELATIONS), and the expression values for all genes quantified (ALL GENES). All expression values are given in nucleotide coverage after normalization to total histone RNA coverage.
(XLSX)
