# Supplementary figures and images for: Drosophila Nipped-B Mutants Model Cornelia de Lange Syndrome in Growth and Behavior
Source: PLoS Genet. 2015 Nov 6;11(11):e1005655. doi: 10.1371/journal.pgen.1005655 (PMC4636142; doi:10.1371/journal.pgen.1005655)

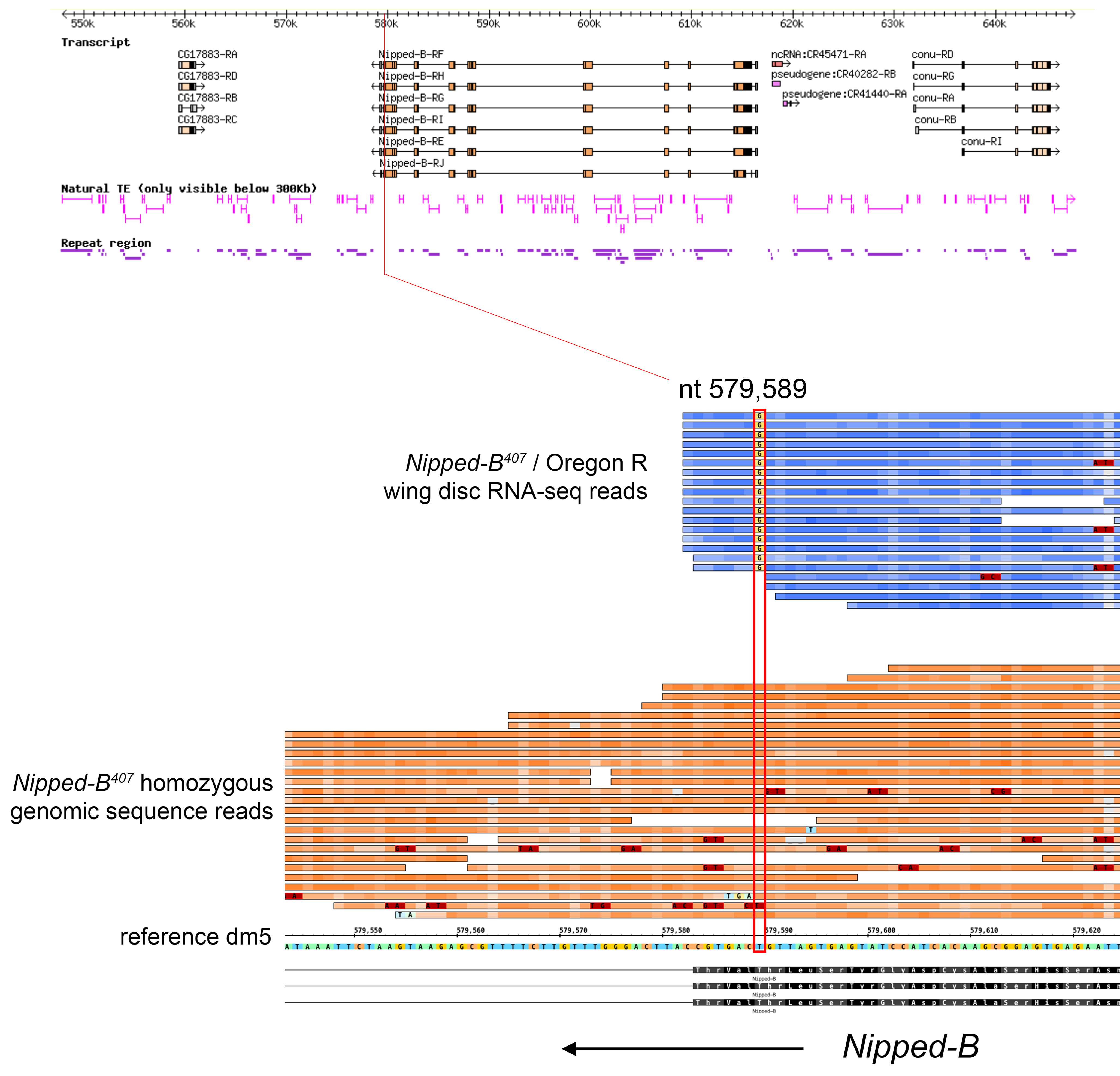

Supplement: S1 Fig — The top diagram shows the location of Nipped-B in centromere-proximal heterochromatin on chromosome 2R, with extensive repeat sequences, including many natural transposable elements (TEs, pink and purple). The Nipped-B gene displays histone H3 lysine 9 trimethylation (H3K9me3) and HP1 occupancy typically associated with heterochromatin. The homozygous Nipped-B 407 genomic sequence shows that nucleotide 579,589 (release 5 April 2006 coordinates) in the second to last exon matches the reference genome (T in plus strand, A in minus strand), while the RNA sequence from Nipped-B 407 / Oregon R wing discs does not, with all aligned reads showing C in the minus strand (G in plus strand). This is a silent mutation in the wobble position of a Thr codon that does not change the amino acid sequence. Thus most of the RNA is not produced by Nipped-B 407, suggesting that is essentially a null allele. No mutations were found in the Nipped-B 407 coding sequences by genomic sequencing. RNA-seq sequence coverage shown in S2 Fig further indicates that there are no alterations in transcription start and termination, or in splicing. Thus Nipped-B 407 is likely a chromosomal rearrangement (insertion, deletion, transposition or inversion) that is hidden by the repetitive sequence nature of the surrounding environment. (TIF) [file pgen.1005655.s001.tif]

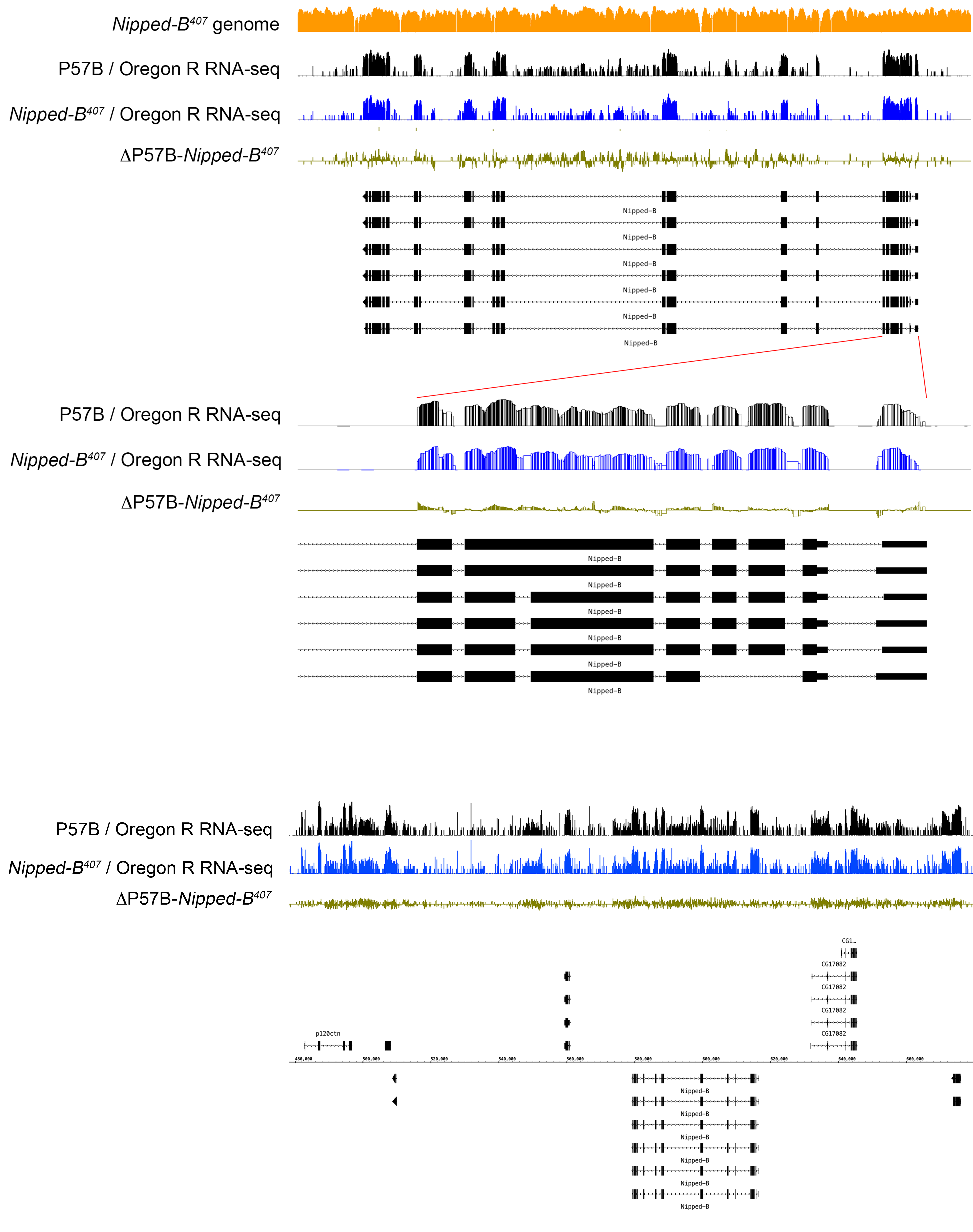

Supplement: S2 Fig — The top shows the sequence coverage (log2 bedgraph) for the minus strand genome sequence of Nipped-B 407 homozygous 2nd instar larvae (orange), and for the minus strand RNA-seq coverage for P57B / Oregon R wing discs (black) and Nipped-B 407 / Oregon R wing discs (blue). The mathematical difference between the P57B and Nipped-B 407 log2 RNA-seq coverage (ΔP57B-Nipped-B 407) is shown in the bottom track (green) to visually compare the transcript patterns in P57B and Nipped-B 407. This subtraction, which calculates fold-differences, reveals that there are no significant changes in coverage across the gene between the P57B control and Nipped-B mutant wing discs, indicating that there are no changes in the transcription start sites, termination sites or splicing. The middle panel shows the 5’ end of the gene, where there is substantial alternative splicing, to show that the splicing pattern is not detectably altered. The bottom panel shows the log2 RNA-seq bedgraphs (combined plus and minus strand transcription) for the entire region surrounding Nipped-B, indicating that transcription of the genes neighboring Nipped-B are not altered by the Nipped-B 407 mutation. Combined with the sequence comparison in S1 Fig, this indicates that the bulk of Nipped-B RNA in Nipped-B 407 / Oregon R wing discs is from the wild-type allele. (TIF) [file pgen.1005655.s002.tif]

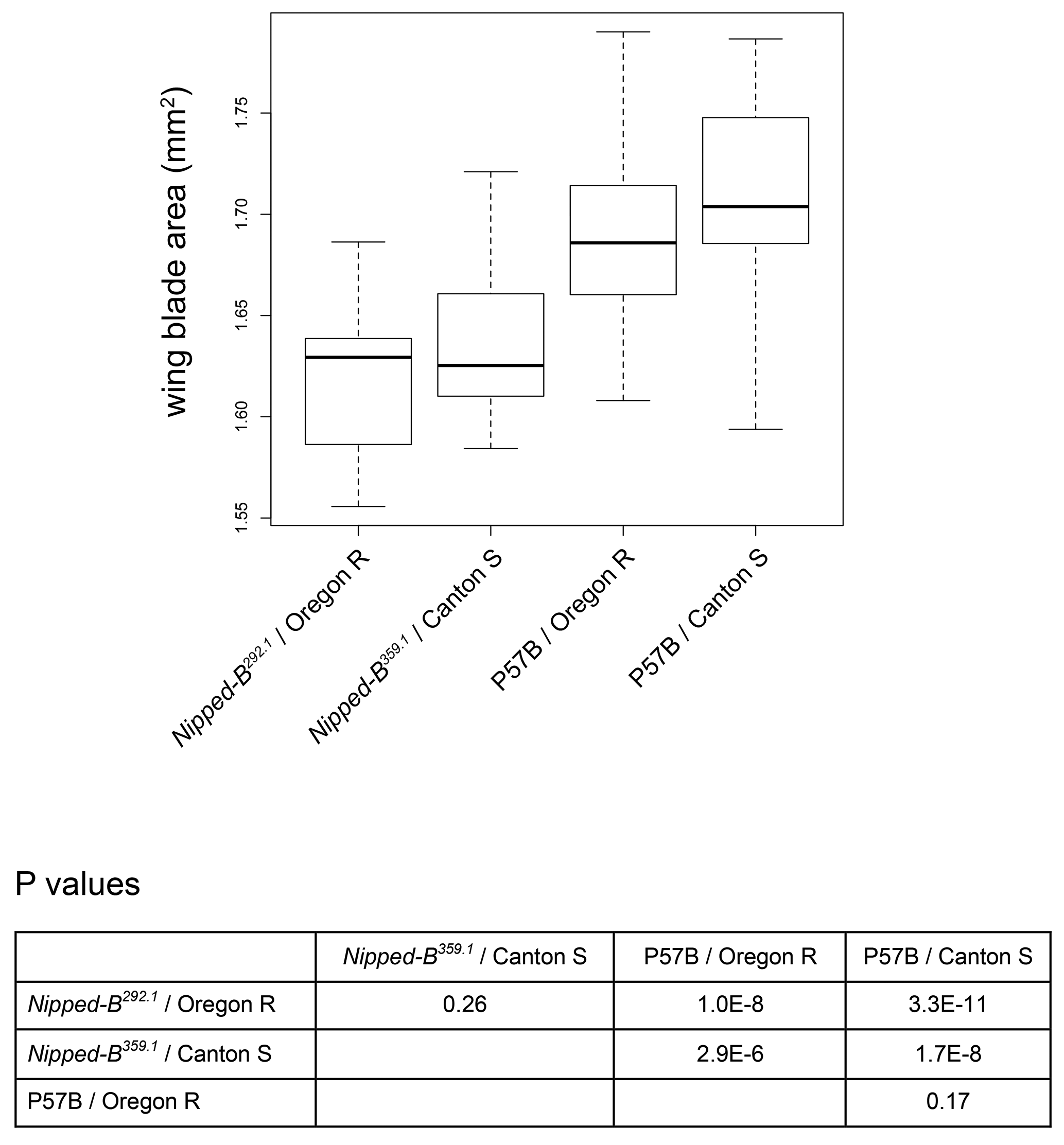

Supplement: S3 Fig — The top panel is a boxplot of the wing blade areas of females of the indicated genotypes, and the bottom panel gives the p values for the indicated comparisons (t test). (TIF) [file pgen.1005655.s003.tif]

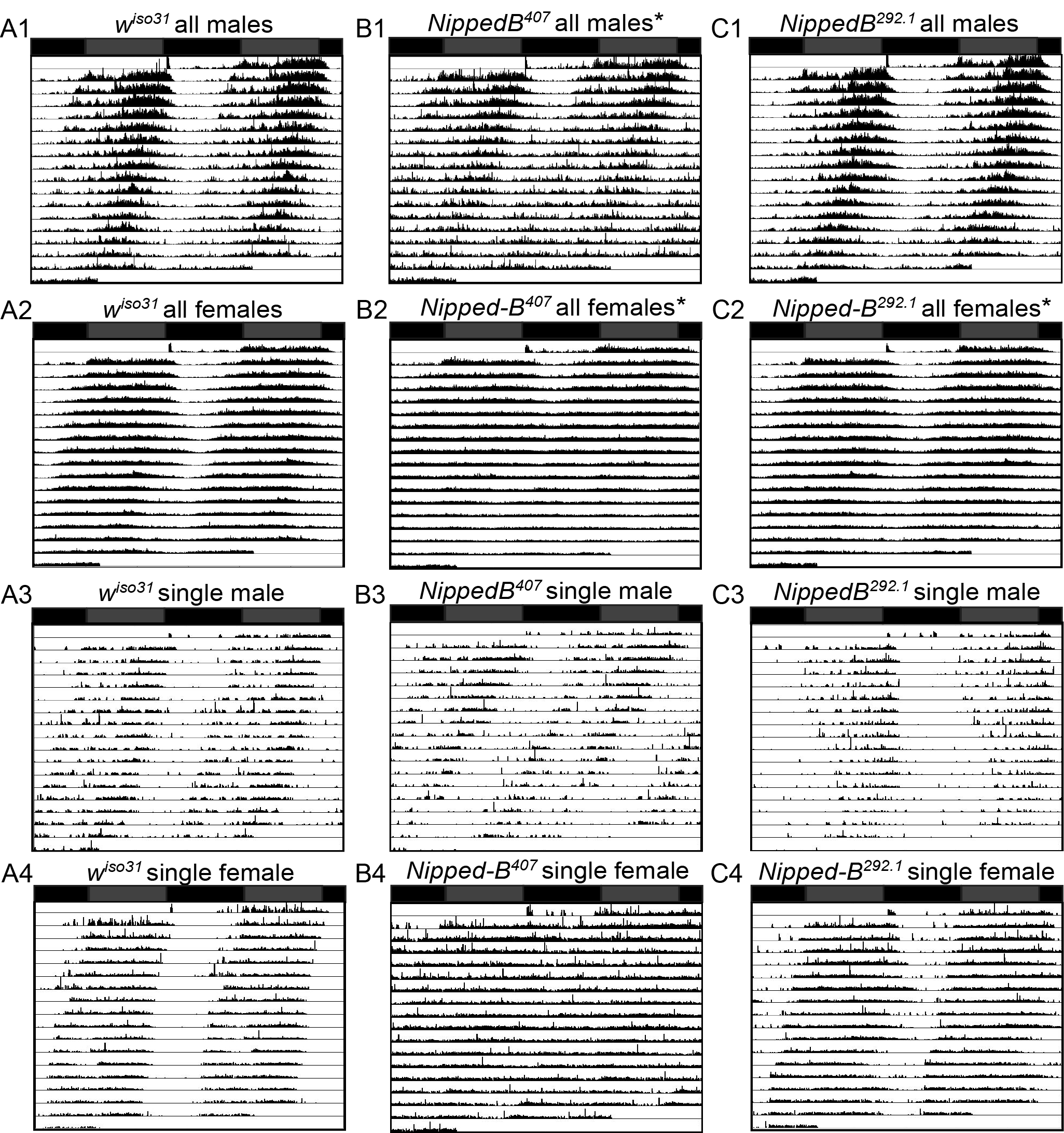

Supplement: S4 Fig — (A1-C1, A2-C2) Average double plotted actograms for w iso31, Nipped-B 291.1, and Nipped-B 407 flies. The numbers of flies in each genotype are described in Fig 8B. The mutants that are significantly different from controls in rhythmicity are denoted by *. (A3-C3, A4-C4) Representative double plotted actograms of individual flies of the indicated genotypes. (TIF) [file pgen.1005655.s004.tif]

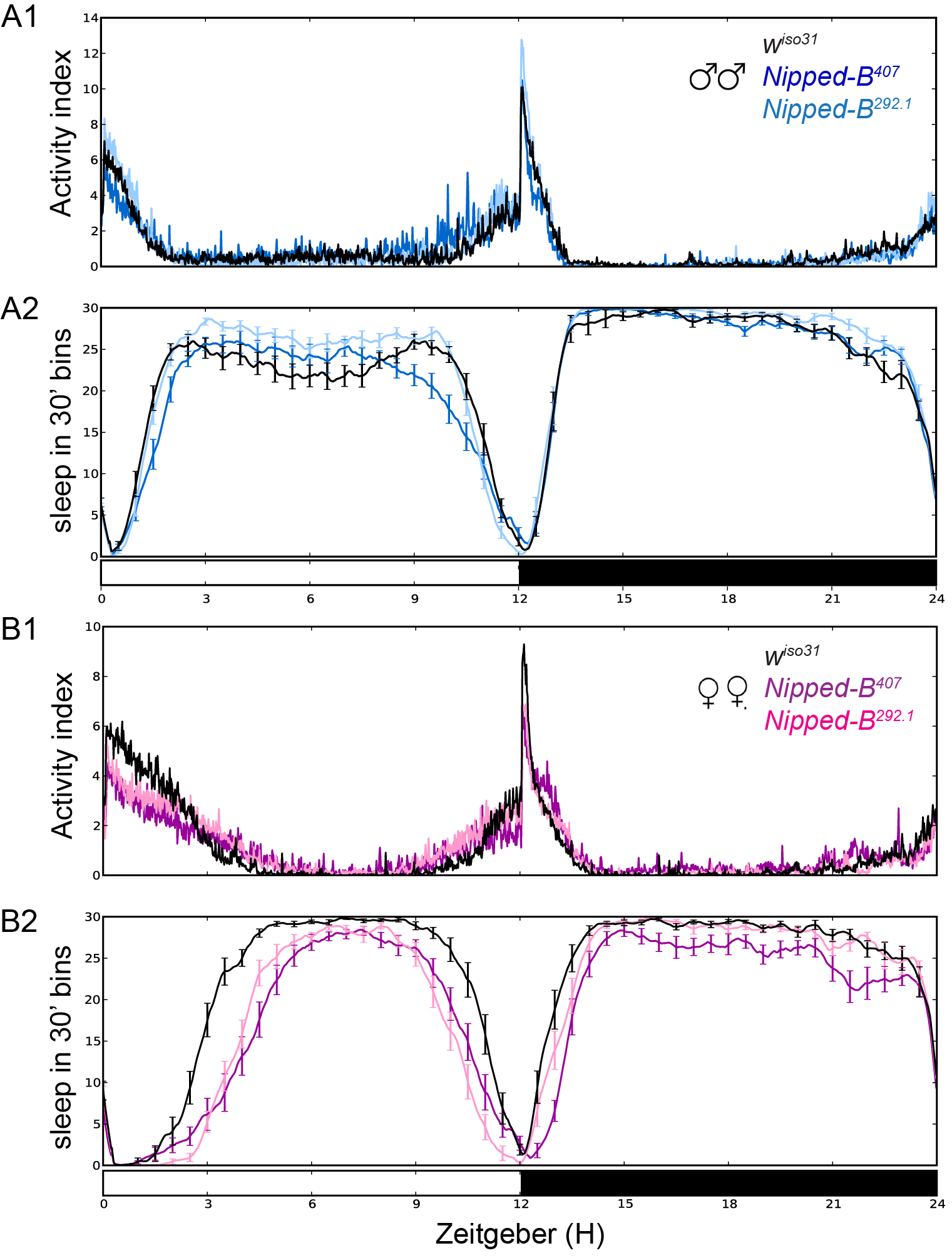

Supplement: S5 Fig — (A1, B1) Average activity patterns of Nipped-B mutant and control males (A1) and females (B1) recorded in 12 hour L/D cycles (15 to 16 flies of each genotype and sex for over six days). The activity of both male and female Nipped-B mutants is comparable to that of wild-type controls, suggesting that they are not hyperactive. (A2, B2) Sleep profiles for males (A2) and females (B2) with standard error bars. These patterns were analyzed in more detail in Fig 7. (TIF) [file pgen.1005655.s005.tif]
